# A stochastic approach for co-evolution process of virus and human immune system

**DOI:** 10.1038/s41598-024-60911-z

**Published:** 2024-05-06

**Authors:** Qura Tul Ain, Jiahao Shen, Peng Xu, Xiaoli Qiang, Zheng Kou

**Affiliations:** 1 School of Mathematics and Information Science, Guangzhou University, Guangzhou, 510006 China; 2 Institute of Computing Science and Technology, Guangzhou University, Guangzhou, 510006 China; 3 School of Computer Science and Cyber Engineering, Guangzhou University, Guangzhou, 510006 China

**Keywords:** Equilibrium, Extinction, Stability analysis, Immune system, Computational biology and bioinformatics, Diseases, Health care, Mathematics and computing

## Abstract

Infectious diseases have long been a shaping force in human history, necessitating a comprehensive understanding of their dynamics. This study introduces a co-evolution model that integrates both epidemiological and evolutionary dynamics. Utilizing a system of differential equations, the model represents the interactions among susceptible, infected, and recovered populations for both ancestral and evolved viral strains. Methodologically rigorous, the model’s existence and uniqueness have been verified, and it accommodates both deterministic and stochastic cases. A myriad of graphical techniques have been employed to elucidate the model’s dynamics. Beyond its theoretical contributions, this model serves as a critical instrument for public health strategy, particularly predicting future outbreaks in scenarios where viral mutations compromise existing interventions.

## Introduction

**Figure 1:**
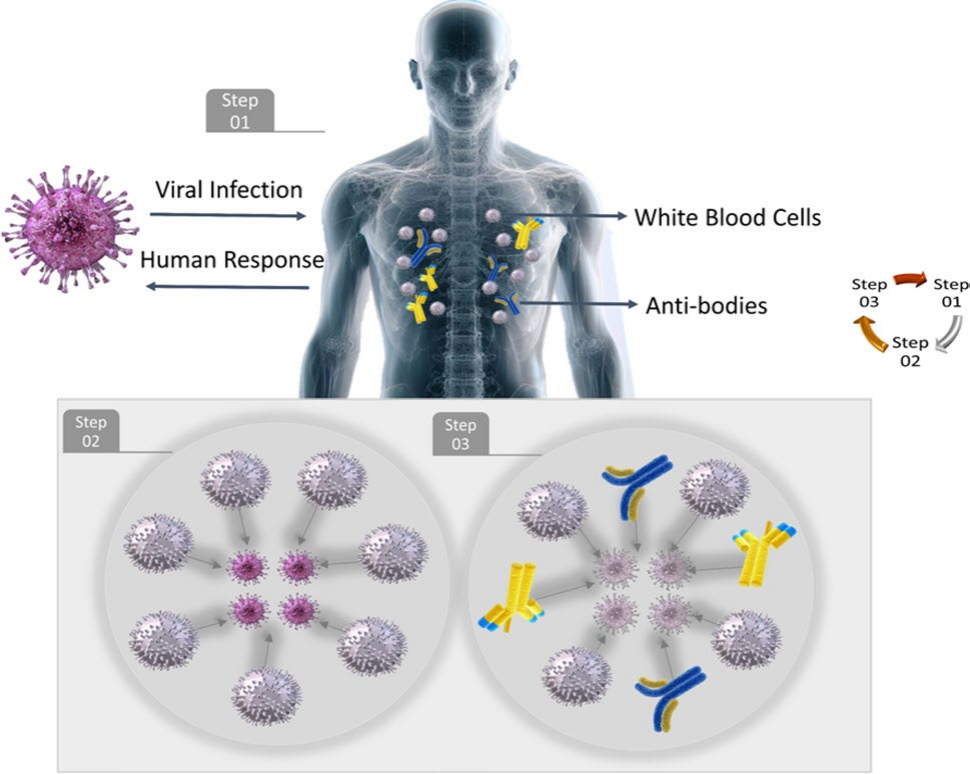
Virus and immune system’s co-evolution process.

Infectious diseases have shaped human history, leading to profound societal and cultural impacts. As our understanding of epidemiology has evolved, so too has the complexity of the models we use to predict and understand disease dynamics. The interplay between pathogens and hosts is a continuous arms race; while pathogens mutate to become more virulent or avoid host immunity, hosts evolve to develop improved resistance or immunity against these pathogens. This cyclical nature of adaptation and counter-adaptation (Fig. 1) has been a crucial element in the evolutionary history of many species, including humans.

In recent years, with the rise of diseases caused by rapidly mutating viruses, there is a heightened interest in understanding not just the dynamics of disease spread, but also how evolution plays a role in these dynamics. Traditional epidemiological models, such as the SIR (Susceptible-Infectious-Recovered) model, focus primarily on disease transmission without accounting for evolutionary changes in the virus or the host. In the landscape of infectious disease modeling, several pivotal works have laid the groundwork for comprehensive understanding. Authors in^1^ discussed a mathematical model accounting for the dynamics of multiple SARS-CoV-2 strains focused on the impact of variants on pandemic trajectories and vaccine response. The study emphasizes the utility of the model in predicting variant rises and informing vaccination strategies.^2^ took a generic approach to mathematical modeling of multi-strain pandemics, while^3^ presented a model that focused on leveraging multiple strains with mutations in the context of COVID-19. Contributions from^4^ incorporated vaccination dynamics into a two-strain model of COVID-19, whereas the work in^5^ emphasized the diverse outcomes among COVID-19 patients. Study in^6^ discussed the impact of reproduction numbers on multiwave spreading dynamics, while^7^ focused on the interplay between innate and adaptive immune responses.^8^ analyzed the host immunological response to adenovirus-based COVID-19 vaccines. Evidences from^9^ brought forth a model assessing the level of cross-immunity between influenza strains.

A research develops a two-strain COVID-19 transmission model addressing the emergence of variants with different transmission dynamics and analyzing the impact of vaccination on one of the strains. It provides a theoretical framework with sufficient conditions for equilibrium stability, calculating the basic reproduction numbers and exploring scenarios for dominant strain establishment^10^. The competition between different SARS-CoV-2 variants in France using a mathematical model to estimate the impact of three variants on the spread of COVID-19, employing data from Geodes and a particle swarm optimization algorithm to estimate the basic reproduction number can be seen in^11^. A fractional model of two-strains covid disease was discussed by^12^.

Collectively, these references provide a rich tapestry of insights into the evolving dynamics of infectious diseases. However, to truly grasp the progression and potential future trajectories of such diseases, one must consider the evolutionary dynamics in tandem with epidemiological ones.

### Motivation

Our study advances the understanding of multiple infection dynamics, building on significant previous research in disease modeling. We resonate with and expand upon the work of researchers like those cited in^13,14^, and^15^, who explored transmission dynamics and sensitivity analysis using fractal-fractional differential operators. Our methodology also draws from^16,17^, who developed fractional models for diseases such as malaria and typhoid fever, underscoring the critical role of analyzing multiple pathogens in epidemiological studies and the importance of understanding their reproduction numbers.

Furthermore, we recognize the impact of stochastic components in modeling diseases, as highlighted in^18^. The incorporation of time delays in stochastic epidemic models introduces complex system behaviors. The research in^19,20^, and^21^ demonstrates how mathematical and statistical approaches can be employed to tackle questions of stochasticity and stability in epidemic models. Additionally,^22^’s work on modeling COVID-19 with fractional order calculus emphasizes the effect of policy measures like isolation and vaccination on disease control, complementing our discussions on the efficacy of public health interventions.

Li and colleagues’ contributions^23,24,25^ shed light on the dynamic behavior of epidemic models, especially regarding bifurcations and chaotic phenomena, offering a valuable backdrop to our findings on oscillatory behaviors near endemic equilibrium.

### Contribution

This work introduces a co-evolution model that seeks to capture this intricate balace between evolving viruses and the evolving human immune response. This model, built upon differential equations, represents the interaction dynamics of susceptible, infected, and recovered populations with both the original and evolved virus strains. By integrating evolutionary considerations into traditional epidemiological models, we aim to provide a more nuanced and comprehensive understanding of disease dynamics in the face of viral mutations and changing host immunities.

### Novelty

Stochastic modeling plays a crucial role in epidemiology for several reasons. It allows researchers to account for the inherent randomness and variability in disease transmission among individuals and communities, reflecting the real-world unpredictability of outbreaks. This is vital for accurately simulating the spread of diseases and assessing potential outcomes under different scenarios. Stochastic models help in estimating the probabilities of different epidemiological events, enabling public health officials to make informed decisions regarding intervention strategies and resource allocation. Through stochastic modeling, epidemiologists can better understand the dynamics of infectious diseases, including the impact of factors like vaccination rates, population density, and social behaviors, thereby improving the effectiveness of disease control and prevention measures.

Beyond its theoretical significance, this model serves as a tool to guide public health interventions, and predict potential future outbreaks, especially in scenarios where rapid viral mutations might render existing treatments or vaccines less effective over time. In the subsequent sections, we will delve into the assumptions underlying the model, the mathematical formulations representing the co-evolution dynamics, and potential applications and implications of the findings derived from this model.

## Model explanation

The model captures the interplay between human immunity and viral evolution. As the virus spreads, the human immune response evolves, leading to a changing transmission rate for the virus. The model can be used to analyze the impact of various interventions.

1 $$\begin{aligned} \begin{aligned} \frac{d\breve{S}_1}{dt}&= \mu - \beta _1 {\breve{S_1}} {\breve{I_1}} - \beta _2 {\breve{S_1}} {\breve{I_2}} + \rho {\breve{R}} - \delta {\breve{S_1}}, \\ \frac{d\breve{S}_2}{dt}&= -\beta _2 {\breve{S_2}} {\breve{I_2}} - \beta _1 {\breve{S_2}} {\breve{I_1}} - \delta {\breve{S_2}}, \\ \frac{d\breve{I}_1}{dt}&= \beta _1 {\breve{S_2}} {\breve{I_1}} - \gamma {\breve{I_1}} - \sigma {\breve{I_1}} - \delta {\breve{I_1}}, \\ \frac{d\breve{I}_2}{dt}&= \beta _2 {\breve{S_1}} {\breve{I_2}} - \gamma {\breve{I_2}} - \sigma {\breve{I_2}} - \delta {\breve{I_2}}, \\ \frac{d\breve{R}}{dt}&= \gamma {\breve{I_1}} + \gamma {\breve{I_2}} - \rho {\breve{R}} - \delta {\breve{R}}. \end{aligned} \end{aligned}$$

with

$$\begin{aligned} \beta _1 = \beta _2 = \beta _0 \times \left( 1 + \alpha \times r \times I \times \left( 1 - \frac{I}{K}\right) \right) . \end{aligned}$$

- ${\breve{S_1}}$ and ${\breve{S_2}}$ represent two subpopulations of the host species that are susceptible to infection by the virus strains ${\breve{I_1}}$ and ${\breve{I_2}}$, respectively.
- ${\breve{R}}$ represents the recovered individuals.
- $\beta _1$ and $\beta _2$ are the transmission rates of the two virus strains
- *r* influences the rate at which the transmission rates ($\beta _1$ and $\beta _2$) change in response to changes in the total number of infected individuals.
- The parameter *r* significantly influences the transmission rates $\beta _1$ and $\beta _2$ for the two virus strains. Specifically, the transmission rates are defined as $\beta _1 = \beta _2 = \beta _0 \times \left( 1 + \alpha \times r \times I \times \left( 1 - \frac{I}{K}\right) \right)$, indicating that the value of *r* directly affects how the total number of infected individuals *I* influences $\beta _1$ and $\beta _2$. An increase in *r* amplifies the effect of changes in *I* on the transmission rates, due to the term $\alpha \times r \times I \times \left( 1 - \frac{I}{K}\right)$. Thus, *r* acts as a modulator of the transmission rates’ sensitivity to the infected population size, playing a crucial role in the disease’s spread dynamics within the host population.
- The parameter $\alpha$ plays a crucial role in the model, acting as a factor within the feedback mechanism that influences the transmission rates $\beta _1$ and $\beta _2$. Its primary function is to modulate the effect of the infected population size on the transmission rates, enabling the model to incorporate dynamic changes in transmission potential that occur as the prevalence of infection within the population changes. The model considers a moderate level of feedback, wherein increases in the infected population proportionally adjust the transmission rates, but not to an extreme degree. This adjustment is critical for accurately modeling the spread of infectious diseases, as it reflects the complex interactions between host behavior, population density, and pathogen transmissibility that can affect the rate at which an infection spreads through a community.
- $\delta$ represents the death rate, $\mu$ is birth rate.
- $\rho$ represents the rate at which recovered individuals lose immunity and move back into the susceptible category.

This models a loss of immunity over time. This work took motivation from recent work on stochastic modeling and infectious diseases models as discussed by^26–32^. The stochastic model is given by

2 $$\begin{aligned} \begin{aligned} d\breve{S}_1(\textrm{t})&= \left[ \mu - \beta _1 {\breve{S_1}} {\breve{I_1}} - \beta _2 {\breve{S_1}} {\breve{I_2}} + \rho {\breve{R}} - \delta {\breve{S_1}} \right] dt + \sigma _1 {\breve{S_1}}(\textrm{t}) d{\mathfrak {D}}_1(\textrm{t}), \\ d\breve{S}_2(\textrm{t})&= \left[ -\beta _2 {\breve{S_2}} {\breve{I_2}} - \beta _1 {\breve{S_2}} {\breve{I_1}} - \delta {\breve{S_2}} \right] dt + \sigma _2 {\breve{S_2}}(\textrm{t}) d{\mathfrak {D}}_2(\textrm{t}), \\ d\breve{I}_1(\textrm{t})&= \left[ \beta _1 {\breve{S_2}} {\breve{I_1}} - \gamma {\breve{I_1}} - \sigma {\breve{I_1}} - \delta {\breve{I_1}} \right] dt + \sigma _3 {\breve{I_1}}(\textrm{t}) d{\mathfrak {D}}_3(\textrm{t}), \\ d\breve{I}_2(\textrm{t})&= \left[ \beta _2 {\breve{S_1}} {\breve{I_2}} - \gamma {\breve{I_2}} - \sigma {\breve{I_2}} - \delta {\breve{I_2}} \right] dt + \sigma _4 {\breve{I_2}}(\textrm{t}) d{\mathfrak {D}}_4(\textrm{t}), \\ d\breve{R}(\textrm{t})&= \left[ \gamma {\breve{I_1}} + \gamma {\breve{I_2}} - \rho {\breve{R}} - \delta {\breve{R}} \right] dt + \sigma _5 {\breve{R}}(\textrm{t}) d{\mathfrak {D}}_5(\textrm{t}). \end{aligned} \end{aligned}$$

The model includes several $\sigma$ parameters representing variability and stochastic effects:

- $\sigma _1$: Represents the variability in the susceptible population $\breve{S_1}$, which arise from fluctuating contact rates or changes in population behavior that affect exposure to the first virus strain.
- $\sigma _2$: Captures the randomness in the second susceptible population $\breve{S_2}$, which is due to similar factors as $\sigma _1$, but with different underlying causes or magnitudes, given that $S_2$ represent a different risk group.
- $\sigma _3$: Reflects the random fluctuations in the number of individuals infected with the first virus strain $\breve{I_1}$, due to variations in the disease’s infectiousness, reporting rates, or response to treatment.
- $\sigma _4$: Pertains to the variability in the infection rate of the second virus strain $\breve{I_2}$, which differ from $\sigma _3$ as the new strain has distinct characteristics, that is, higher transmissibility.
- $\sigma _5$: Represents stochastic factors affecting the recovered population $\breve{R}$, such as differential rates of loss of immunity or the impact of interventions that are not consistent across the entire population.

Each $\sigma$ parameter is paired with a corresponding Brownian motion term, which mathematically represents the random ’noise’ contributing to the fluctuations in each compartment over time. By including these stochastic terms, the model becomes a set of stochastic differential equations (SDEs), providing a more nuanced and realistic simulation of the epidemiological process, which can now capture both the average trends and the variability around those averages. The following assumptions underlie the model:

- Every parameter within the system is a nonnegative, positive real number.
- The transmission rate of the virus can change based on the proportion of the infected population.
- Immunity to one strain does not confer immunity to the evolved strain.

## Qualitative analysis

### Definition 1

A system of differential equations is said to be Lipschitz continuous with respect to a variable $x$ if there exists a constant $L$ such that for any state variables $x$ and $y$ in a domain $D$, then

$$\begin{aligned} ||f(x) - f(y)|| \le L ||x - y||. \end{aligned}$$

### Lemma 1

Consider two solutions, $({\breve{S_1}}, {\breve{S_2}}, {\breve{I_1}}, {\breve{I_2}}, {{\tilde{R}}})$ and $({\breve{S_1}}', {\breve{S_2}}', {\breve{I_1}}', {\breve{I_2}}', {{\tilde{R}}}')$, of the stochastic system. Then, for each equation in the system, the difference between the rates of change for the two solutions is bounded by a constant times the difference between the solutions.

### Proof

Taking equation for ${\breve{S_1}}$,

$$\begin{aligned} \bigg | \bigg (&\mu - \beta _1 \breve{S_1} \breve{I_1} - \beta _2 \breve{S_1} \breve{I_2} + \rho {\tilde{R}} - \delta \breve{S_1}\bigg ) \\&- \bigg (\mu - \beta _1 \breve{S_1}' \breve{I_1}' - \beta _2 \breve{S_1}' \breve{I_2}' + \rho {\tilde{R}}' - \delta \breve{S_1}'\bigg ) \bigg |, \\ \le&\,\,\bigg | \beta _1 (\breve{S_1} \breve{I_1} - \breve{S_1}' \breve{I_1}') + \beta _2 (\breve{S_1} \breve{I_2} - \breve{S_1}' \breve{I_2}') + \rho ({\tilde{R}} - {\tilde{R}}') + \delta (\breve{S_1} - \breve{S_1}') \bigg |, \\ \le&\,\,\beta _1 (|\breve{S_1} - \breve{S_1}'|\cdot |\breve{I_1}| + |\breve{S_1}|\cdot |\breve{I_1} - \breve{I_1}'|) \\&+ \beta _2 (|\breve{S_1} - \breve{S_1}'|\cdot |\breve{I_2}| + |\breve{S_1}|\cdot |\breve{I_2} - \breve{I_2}'|) \\&+ \rho |{\tilde{R}} - {\tilde{R}}'| + \delta |\breve{S_1} - \breve{S_1}'|. \end{aligned}$$

We can set,

$$\begin{aligned} L = \beta _1 \left( |{\breve{I_1}}| + |{\breve{S_1}}|\right) + \beta _2 \left( |{\breve{I_2}}| + |{\breve{S_1}}|\right) + \rho + \delta . \end{aligned}$$

as an upper bound.

Repeating this process for the other equations, we can identify similar constants for each one. The largest of these constants then serves as the Lipschitz constant $L$ for the whole system. $\square$

### Definition 2

A system is considered bounded if, for all solutions $x(\textrm{t})$ of the system and some positive constant $M$, the inequality $||x(\textrm{t})|| \le M$ holds for all $\textrm{t}$.

### Theorem 1

The stochastic system, given appropriate initial conditions, exhibits bounded behavior.

### Proof

Consider the first equation for ${\breve{S_1}}$:

$$\begin{aligned} \frac{dS_1}{dt} = \mu - \beta _1 {\breve{S_1}} {\breve{I_1}} - \beta _2 {\breve{S_1}} {\breve{I_2}} + \rho {{\tilde{R}}} - \delta {\breve{S_1}} + \xi _1(\textrm{t}). \end{aligned}$$

Given that populations cannot be negative, the loss terms ($\beta _1 {\breve{S_1}} {\breve{I_1}}$, $\beta _2 {\breve{S_1}} {\breve{I_2}}$, $\delta {\breve{S_1}}$) ensure that ${\breve{S_1}}$ does not grow unbounded. Therefore, the deterministic part of the system is bounded. $\square$

### Theorem 2

Given boundedness and Lipschitz continuity, there exists a unique solution to the stochastic model for all time.

### Proof

For a system of stochastic differential equations (SDEs) of the form:

$$\begin{aligned} dX(\textrm{t}) = f(X(\textrm{t})) dt + g(X(\textrm{t})) dW(\textrm{t}). \end{aligned}$$

where $dW(\textrm{t})$ represents the Wiener process, the existence and uniqueness of its solution is ensured if:

- The coefficients $f$ and $g$ are bounded.
- The system is Lipschitz continuous.

We’ve shown that the system is Lipschitz continuous and bounded. Hence, we conclude that there exists a unique solution to the SDEs of our stochastic model for all time. $\square$

## Equilibrium analysis

The variational matrix is obtained by linearizing the system of differential equations around the equilibrium. If we denote the equilibria as $({{\breve{S_{1e}}}}, {{\breve{S_{2e}}}}, {{\breve{I_{1e}}}}, {{\breve{I_{2e}}}}, {{\tilde{R}}}_e)$, the variational matrix ${\mathcal {V}}$ at this equilibrium is given by the Jacobian:

$$\begin{aligned} {\mathcal {V}} = \begin{pmatrix} -\beta _1 {{\breve{I_{1e}}}} - \beta _2 {{\breve{I_{2e}}}} - \delta &{} -\beta _1 {{\breve{S_{1e}}}} - \beta _2 {{\breve{S_{1e}}}} &{} \mu - \beta _1 {{\breve{S_{1e}}}} &{} \mu - \beta _2 {{\breve{S_{1e}}}} &{} \rho \\ -\beta _2 {{\breve{I_{2e}}}} - \beta _1 {{\breve{I_{1e}}}} &{} -\beta _2 {{\breve{S_{2e}}}} - \beta _1 {{\breve{S_{2e}}}} &{} \beta _1 {{\breve{S_{2e}}}} &{} \beta _2 {{\breve{S_{2e}}}} &{} 0 \\ \beta _1 {{\breve{S_{2e}}}} &{} \beta _1 {{\breve{I_{1e}}}} &{} -\gamma - \sigma - \delta &{} 0 &{} 0 \\ \beta _2 {{\breve{S_{1e}}}} &{} \beta _2 {{\breve{I_{2e}}}} &{} 0 &{} -\gamma - \sigma - \delta &{} 0 \\ \gamma &{} \gamma &{} -\rho &{} -\rho &{} -\delta \\ \end{pmatrix}. \end{aligned}$$

### $E_1(0,0,0,0,0)$

Given the variational matrix at the equilibrium ${\mathfrak {E}}_1(0,0,0,0)$, we have ${{\breve{I_{1e}}}} = {{\breve{I_{2e}}}} = {{\breve{S_{1e}}}} = {{\breve{S_{2e}}}} = {\breve{R_{e}}} =0$.

$$\begin{aligned} {\mathcal {V}} = \begin{pmatrix} -\delta &{} 0 &{} \mu &{} \mu &{} \rho \\ 0 &{} 0 &{} 0 &{} 0 &{} 0 \\ 0 &{} 0 &{} -\gamma - \sigma - \delta &{} 0 &{} 0 \\ 0 &{} 0 &{} 0 &{} -\gamma - \sigma - \delta &{} 0 \\ \gamma &{} \gamma &{} -\rho &{} -\rho &{} -\delta \\ \end{pmatrix}. \end{aligned}$$

The eigenvalues of the variational matrix ${\mathcal {V}}$ at the equilibrium point ${\mathfrak {E}}_1(0,0,0,0,0)$ are $-\delta$, $0$, $-\gamma - \sigma - \delta$,$-\gamma - \sigma - \delta$,$-\delta$. Therefore, the equilibrium ${\mathfrak {E}}_1(0,0,0,0,0)$ is semi-stable based on the eigenvalues of the variational matrix.

#### Theorem 3

The stability point $E_1(0,0,0,0,0)$ exhibits local asymptotic stability provided that $\delta > 0$, $\gamma + \sigma + \delta > 0$.

### ${E}_2(1,0,0,0,0)$

Given the equilibrium ${\mathfrak {E}}_1(1,0,0,0,0)$ where ${{\breve{I_{1e}}}} = 1$, ${{\breve{I_{2e}}}} = {{\breve{S_{1e}}}} = {{\breve{S_{2e}}}} = {\breve{R_{e}}} = 0$, substituting in the values for the equilibrium, we get,

$$\begin{aligned} {\mathcal {V}}_{{\mathfrak {E}}_1} = \begin{pmatrix} -\beta _1 - \delta &{} 0 &{} \mu &{} \mu &{} \rho \\ -\beta _1 &{} 0 &{} 0 &{} 0 &{} 0 \\ 0 &{} \beta _1 &{} -\gamma - \sigma - \delta &{} 0 &{} 0 \\ 0 &{} 0 &{} 0 &{} -\gamma - \sigma - \delta &{} 0 \\ \gamma &{} \gamma &{} -\rho &{} -\rho &{} -\delta \\ \end{pmatrix}. \end{aligned}$$

The eigenvalues are $-\beta _1 - \delta$,$0$,$-\gamma - \sigma - \delta$,$-\gamma - \sigma - \delta$,$-\delta$.

#### Theorem 4

The stability point $E_2(1,0,0,0,0)$ exhibits local asymptotic stability provided that $\beta _1 + \delta > 0$, $\gamma + \sigma + \delta > 0$, $\delta > 0$.

**The remaining part of equilibrium analysis can be found in** Appendix section 12.1.

### Endemic equilibrium

The asymptotic solution relies heavily on the basic reproduction number $R_0$ of the disease, which is the expected number of cases directly generated by one case in a population where all individuals are susceptible to infection^33^. The presence of an endemic equilibrium depends on various factors, including the basic reproduction number $R_0$. The Jacobian matrix $J$ of the system at the DFE (disease free equilibrium) is given by:

$$\begin{aligned} J = \begin{bmatrix} \mu - \delta &{} 0 &{} -\beta _1 {\breve{S_1}} &{} -\beta _2 {\breve{S_1}} &{} \rho \\ 0 &{} -\delta &{} -\beta _2 {\breve{S_2}} &{} -\beta _1 {\breve{S_2}} &{} 0 \\ 0 &{} 0 &{} -\gamma - \sigma - \delta &{} 0 &{} 0 \\ 0 &{} 0 &{} 0 &{} -\gamma - \sigma - \delta &{} 0 \\ 0 &{} 0 &{} \gamma &{} \gamma &{} -\rho - \delta \\ \end{bmatrix}. \end{aligned}$$

The next-generation matrix, $K$, is given by the product of two matrices, $F$ and $V^{-1}$, For our system, the matrix $F$ is

$$\begin{aligned} F = \begin{bmatrix} \beta _1 {\breve{S_2}} &{} 0 \\ 0 &{} \beta _2 {\breve{S_1}} \\ \end{bmatrix}. \end{aligned}$$

The matrix $V$ is

$$\begin{aligned} V = \begin{bmatrix} \gamma + \sigma + \delta &{} 0 \\ 0 &{} \gamma + \sigma + \delta \\ \end{bmatrix}. \end{aligned}$$

The inverse is

$$\begin{aligned} V^{-1} = \begin{bmatrix} \frac{1}{\gamma + \sigma + \delta } &{} 0 \\ 0 &{} \frac{1}{\gamma + \sigma + \delta } \\ \end{bmatrix}. \end{aligned}$$

The next-generation matrix $K$ is

$$\begin{aligned} K = F \times V^{-1} = \begin{bmatrix} \frac{\beta _1 {\breve{S_2}}}{\gamma + \sigma + \delta } &{} 0 \\ 0 &{} \frac{\beta _2 {\breve{S_1}}}{\gamma + \sigma + \delta } \\ \end{bmatrix}. \end{aligned}$$

Thus, ${R}_0$ is the maximum of the two diagonal entries.

$$\begin{aligned} {R}_0 = \max \left( \frac{\beta _1 {\breve{S_2}}}{\gamma + \sigma + \delta }, \frac{\beta _2 {\breve{S_1}}}{\gamma + \sigma + \delta } \right) . \end{aligned}$$

For ${R}_{01}$ for ${\breve{I_1}}$ is $\frac{\beta _2 {\breve{S_1}}}{\gamma + \sigma + \delta }$, For $R_{02}$ for ${\breve{I_2}}$ is $\frac{\beta _1 {\breve{S_2}}}{\gamma + \sigma + \delta }$. To determine the endemic equilibrium, we will evaluate the following basic reproduction numbers,

- ${\textbf{R}}_{{I}_{1}}$ represents the mean count of recent infection cases attributed to ${I}_{1}$.
- ${\textbf{R}}_{{I}_{2}}$ symbolizes the average count of new infection cases ascribed to ${I}_{2}$.

The system’s critical parameter can be expressed as,

3 $$\begin{aligned} {\textbf{R}}_{0} = \max \left( {\textbf{R}}_{{I}_{1}}, {\textbf{R}}_{{I}_{2}}\right) . \end{aligned}$$

Beyond the equilibria highlighted earlier, the model’s endemic equilibrium is realized when,

4 $$\begin{aligned} \min \left( {\textbf{R}}_{{I}_{1}}, {\textbf{R}}_{{I}_{2}}\right) > 1. \end{aligned}$$

#### Theorem 5

There exists a unique endemic equilibrium whenever $R_0 > 1$.

**Proof can be found in** Appendix section 12.2.

## Stochastic analysis

Suppose a probability domain represented as $( \Phi , {\mathcal {G}}, {\mathbb {Q}} )$ containing a Wiener process (or Brownian motion) represented as ${\mathcal {W}} = \left\{ {\mathcal {W}}_{\pi }, {\mathcal {G}}_{\pi }^{{\mathcal {W}}}, \pi > 0 \right\}$. The associated filtration is given by $({\mathcal {G}}_\pi , \pi > 0)$. Let

5 $$\begin{aligned} \textrm{d} {\mathbb {Z}}(\pi ) = {\textbf{y}}(\pi , {\mathbb {Z}}(\pi )) \textrm{d} {\mathcal {W}}(\pi ) + {\textbf{v}}(\pi , {\mathbb {Z}}(\pi )) \textrm{d} \pi , \end{aligned}$$

as the governing stochastic differential equation. The function ${\textbf{v}}(\pi , {\mathbb {Z}}(\pi ))$ maps from $[0, \infty ) \times {\mathbb {R}}^{e}$ to ${\mathbb {R}}^{e}$. ${\textbf{y}}(\pi , {\mathbb {Z}}(\pi ))$ is considered to be an $p \times q$ matrix. In this scenario, both ${\textbf{y}}$ and ${\textbf{v}}$ satisfy Lipschitz conditions.

Let’s introduce ${\textbf{K}}$ as the differential operator for the system described in equation 5 ,

$$\begin{aligned} {\textbf{K}} = \sum _{k=1}^{e} {\textbf{v}}_{k}(\pi ) \frac{\partial }{\partial w_{k}} + \frac{\partial }{\partial \pi } + \frac{1}{2} \sum _{k, l^{*}=1}^{e} \left[ {\textbf{y}}^{T}(w, \pi ) {\textbf{y}}(w, \pi ) \right] _{kl} \frac{\partial ^{2}}{\partial w_{k} \partial w_{l^{*}}}. \end{aligned}$$

Applying operator ${\textbf{K}}$ on a function $\Psi$, where $\Psi \in {\mathbb {C}}^{2,1} \left( {\mathbb {R}}^{e} \times [s_0, \infty ) ; {\mathbb {R}}_+ \right)$, we obtain,

6 $$\begin{aligned} {\textbf{K}} \Psi (w, \pi ) = \Psi _{\pi }(w, \pi ) + \Psi _{w}(w, \pi ) {\textbf{v}}(w, \pi ) + \frac{1}{2} {\text {trace}} \left[ f^{T}(w, \pi ) {\textbf{y}}(w, \pi ) \right] . \end{aligned}$$

### Lemma 2

^34^ Suppose ${\textbf{v}} \in {\mathbb {D}}[[0, \infty ] \times \Theta ,(0, \infty )]$. Our aim is to determine $\kappa _{0}$ and $\kappa > 0$ such that,

$$\begin{aligned} \log {\textbf{v}}({\textrm{t}}) \ge \kappa \textrm{t} - \kappa _{0} \int _{0}^{\textrm{t}} {\textbf{v}}(s) \textrm{d} s + {\textbf{V}}({\textrm{t}}) \text { a.s.}. \end{aligned}$$

Given $\textrm{t} \ge 0$ and ${\textbf{V}} \in ({\mathbb {D}}[[0, \infty ] \times \Theta ,(0, \infty )])$ satisfying $\lim _{\textrm{t} \rightarrow \infty } \frac{{\textbf{V}}({\textrm{t}})}{\textrm{t}} = 0$ a.s., we obtain,

$$\begin{aligned} \lim _{\textrm{t} \rightarrow \infty } \langle {\textbf{v}}({\textrm{t}}) \rangle \ge \frac{\kappa }{\kappa _{0}} \text { a.s.}. \end{aligned}$$

### Global existence

#### Theorem 6

The system has a bounded solution.

For the stochastic system with ${\breve{S_1}}, {\breve{S_2}}, {\breve{I_1}}, {\breve{I_2}}, R$, we define ${\varvec{\Xi }}$ as,

$$\begin{aligned} {\varvec{\Xi }} = \left\{ ({\breve{S_1}}({\textrm{t}}), {\breve{S_2}}({\textrm{t}}), {\breve{I_1}}({\textrm{t}}), {\breve{I_2}}({\textrm{t}}), R({\textrm{t}})) \in {\mathbb {R}}^5_+ \Bigg | \sum _{i=1}^{5} X_i({\textrm{t}}) \le \frac{\varvec{\nu }}{{\epsilon }} \right\} , \end{aligned}$$

where $X_i$ is the corresponding compartment. It remains to prove that ${\varvec{\Xi }}$ adheres to the a.s., invariance principle.

**Proof can be found in** Appendix section 12.3.

#### Theorem 7

${\varvec{\Xi }}$ adheres to the almost sure invariance principle for model $(1)$.

**Proof can be found in** Appendix section 12.4.

#### Theorem 8

For $\left( {\breve{S_1}}(0), {\breve{S}}_{2}(0), {\breve{I}}_{1}(0), {\breve{I}}_{2}(0), \breve{R}(0)\right) \in \Xi$, the system (1) has unique and positive solution almost surely.

**Proof can be found in** Appendix section 12.5.

## Extinction

### Lemma 3

Consider $({\breve{S_1}}({\textrm{t}}),{\breve{S_2}}({\textrm{t}}), {\breve{I_1}}(({\textrm{t}}),{\breve{I_2}}({\textrm{t}}), \breve{R}({\textrm{t}}))$ as the solutions of the model (1) provided initial values $({\breve{S_1}}(0),{\breve{S_2}}(0), {\breve{I_1}}(0), {\breve{I_2}}(0), \breve{R}(0)) \in \Xi$, then

$$\begin{aligned} \lim _{\textrm{t}\rightarrow \infty } \frac{{\breve{S_1}}({\textrm{t}})+{\breve{S_2}}({\textrm{t}})+{\breve{I_1}}({{\textrm{t}}})+{\breve{I_2}}({{\textrm{t}}})+\breve{R}({{\textrm{t}}})}{{\textrm{t}}}=0 ~~~~a.s, \\ \begin{aligned} \lim _{\textrm{t}\rightarrow \infty }\int _{0}^{{\textrm{t}}}\frac{{\breve{S_1}}(r) d{\mathfrak {D}}_{1}(r)}{{\textrm{t}}}=0 \text { a.s},\\ \lim _{\textrm{t}\rightarrow \infty }\int _{0}^{{\textrm{t}}}\frac{{\breve{S_2}}(r) d{\mathfrak {D}}_{2}(r)}{{\textrm{t}}}=0 \text { a.s},\\ \lim _{\textrm{t}\rightarrow \infty }\int _{0}^{{\textrm{t}}}\frac{{\breve{I_1}}(r) d{\mathfrak {D}}_{3}(r)}{{\textrm{t}}}=0 \text { a.s},\\ \lim _{\textrm{t}\rightarrow \infty }\int _{0}^{{\textrm{t}}}\frac{{\breve{I_2}}(r) d{\mathfrak {D}}_{4}(r)}{{\textrm{t}}}=0 \text { a.s}.\\ \lim _{\textrm{t}\rightarrow \infty }\int _{0}^{{\textrm{t}}}\frac{\breve{R}(r) d{\mathfrak {D}}_{4}(r)}{{t}}=0 \text { a.s}.\\ \end{aligned} \end{aligned}$$

We take

$$\begin{aligned} \begin{aligned} {\textbf{R}}^{s}_{1}&={\textbf{R}}_{1}-\frac{1}{2}\frac{\varrho _{3}^{2}}{\left( b_{1}\right) }, \\ {\textbf{R}}^{s}_{2}&={\textbf{R}}_{2}-\frac{1}{2}\frac{\varrho _{4}^{3}}{\left( b_{2}\right) }. \end{aligned} \end{aligned}$$

### Theorem 9

Let ${\breve{S_1}}({\textrm{t}}),{\breve{S_2}}({\textrm{t}}), {\breve{I_1}}({\textrm{t}}),{\breve{I_2}}({\textrm{t}}), \breve{R}({\textrm{t}})$ be the solution of the model with initial values

$({\breve{S_1}}(0),{\breve{S_2}}(0), {\breve{I_1}}(0), {\breve{I_2}}(0), \breve{R}(0)) \in \Xi$. The disease go extinct almost surely, if ${\textbf{R}}^{s}_{1}<1$ .

$$\begin{aligned} \lim \limits _{\textrm{t} \rightarrow +\infty } {\breve{I_1}}({\textrm{t}})=0, \end{aligned}$$

and

$$\begin{aligned} \lim \limits _{\textrm{t} \rightarrow +\infty } {\breve{I_2}}({\textrm{t}})=0. \end{aligned}$$

### Proof

See appendix section.

## Analysis of the population dynamics

The following values in Table 1 has been used for numerical simulation. These values are based on theoretical studies and empirical findings^35–38^

**Table 1:** Initial and parametric values.

Parameter	Definition	Value	Source
$\breve{S}_{1_0}$	Initial Susceptible Population 1	0.475	Estimated
$\breve{S}_{2_0}$	Initial Susceptible Population 2	0.475	Estimated
$\breve{I}_{1_0}$	Initial Infected Population 1	0.01	Estimated
$\breve{I}_{2_0}$	Initial Infected Population 2	0.05	Estimated
$\breve{R}_{0}$	Initial Recovered Population	0.0	Estimated
$\beta _0$	Baseline Transmission Rate	0.5	Estimated
$\alpha$	Modulation Factor for Transmission Rate	0.1	Estimated
*r*	Modulation Rate for $\beta _1$ and $\beta _2$	1.0	Estimated
*K*	Carrying Capacity for Infected Population	1.0	Estimated
$\gamma$	Recovery Rate	0.1	Estimated
$\mu$	Birth Rate	0.02	Estimated
$\delta$	Death Rate	0.01	Estimated
$\sigma$	Waning Immunity	0.01	Estimated
$\rho$	Reinfection rate	0.005	Estimated

### Time series

The Euler-Maruyama method for a stochastic differential equation (SDE) of the form

$$\begin{aligned} dX_t = a(X_t, t) dt + b(X_t, t) dW_t, \end{aligned}$$

is given by the following recursive formula,

$$\begin{aligned} X_{n+1} = X_n + a(X_n, t_n) \Delta t + b(X_n, t_n) \Delta W_n, \end{aligned}$$

where $a(X_t, t)$ is the drift coefficient, $b(X_t, t)$ is the diffusion coefficient, and $\Delta W_n$ is the Wiener process increment approximated by $\sqrt{\Delta t} \cdot N(0, 1)$. The Euler-Maruyama approximation of the given stochastic model is,

$$\begin{aligned} \breve{S}_1^{(n+1)}&= \breve{S}_1^{(n)} + \left[ \mu - \beta _1 \breve{S}_1^{(n)} \breve{I}_1^{(n)} - \beta _2 \breve{S}_1^{(n)} \breve{I}_2^{(n)} + \rho \breve{R}^{(n)} - \delta \breve{S}_1^{(n)} \right] \Delta t + \sigma _1 \breve{S}_1^{(n)} \Delta {\mathfrak {D}}_1^{(n)}, \\ \breve{S}_2^{(n+1)}&= \breve{S}_2^{(n)} + \left[ -\beta _2 \breve{S}_2^{(n)} \breve{I}_2^{(n)} - \beta _1 \breve{S}_2^{(n)} \breve{I}_1^{(n)} - \delta \breve{S}_2^{(n)} \right] \Delta t + \sigma _2 \breve{S}_2^{(n)} \Delta {\mathfrak {D}}_2^{(n)}, \\ \breve{I}_1^{(n+1)}&= \breve{I}_1^{(n)} + \left[ \beta _1 \breve{S}_2^{(n)} \breve{I}_1^{(n)} - \gamma \breve{I}_1^{(n)} - \sigma \breve{I}_1^{(n)} - \delta \breve{I}_1^{(n)} \right] \Delta t + \sigma _3 \breve{I}_1^{(n)} \Delta {\mathfrak {D}}_3^{(n)}, \\ \breve{I}_2^{(n+1)}&= \breve{I}_2^{(n)} + \left[ \beta _2 \breve{S}_1^{(n)} \breve{I}_2^{(n)} - \gamma \breve{I}_2^{(n)} - \sigma \breve{I}_2^{(n)} - \delta \breve{I}_2^{(n)} \right] \Delta t + \sigma _4 \breve{I}_2^{(n)} \Delta {\mathfrak {D}}_4^{(n)}, \\ \breve{R}^{(n+1)}&= \breve{R}^{(n)} + \left[ \gamma \breve{I}_1^{(n)} + \gamma \breve{I}_2^{(n)} - \rho \breve{R}^{(n)} - \delta \breve{R}^{(n)} \right] \Delta t + \sigma _5 \breve{R}^{(n)} \Delta {\mathfrak {D}}_5^{(n)}. \end{aligned}$$

- The susceptible populations, represented by ${\breve{S_1}}$ and $\breve{S}_2$, manifest a declining trend in Fig. 3. This reduction is indicative of the individuals transitioning to the infected categories, depleting the pool of individuals that can potentially be infected.
- The infected categories, represented by ${\breve{I_1}}$ and ${\breve{I_2}}$, show a typical infectious disease trajectory, an initial rise as the disease spreads through the susceptible population, a peak representing the maximum number of concurrent infections, and a subsequent decline as individuals recover or die.
- The $\breve{R}$ category, which denotes recovered individuals, exhibits an increasing trend, reflecting the accumulation of individuals who have gained immunity post-infection.

**Figure 2:**
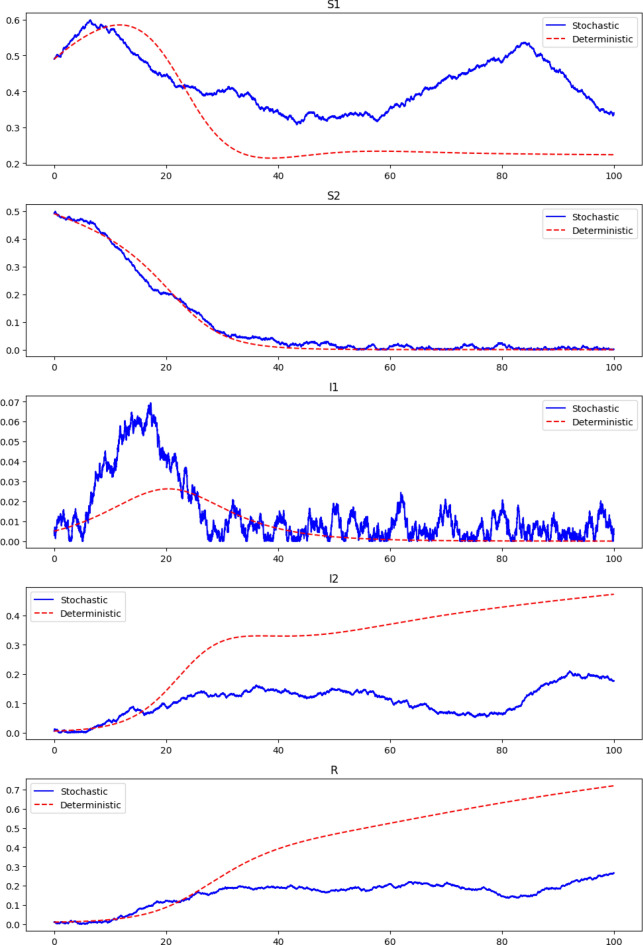
Stochastic VS deterministic time series.

In Fig. 2, the deterministic lines depict an expected trajectory of populations, revealing trends of decline in susceptible classes, a peak in infections, and a rise in recoveries. In contrast, the stochastic lines mirror these general trends but with evident fluctuations, representing real-world variability due to unpredictable factors. Specifically, while susceptibles for both ancestral and evolved strains decrease over time, the evolved strain depletes the susceptible pool more rapidly. The number of infections shows varied dynamics, with the ancestral strain presenting a rapid rise and decline and the evolved strain maintaining a more consistent infection rate.

The recovered population steadily increases over time. The disparities between deterministic and stochastic representations underscore the importance of factoring in real-world randomness alongside average predictions in infectious disease modeling.

${\breve{I_1}}$ is less virulent or less successful at sustaining its spread compared to ${\breve{I_2}}$ given that ${\breve{I_1}}$ dies out, while ${\breve{I_2}}$ reaches a steady state. ${\breve{S_1}}$ and $\breve{S}_2$ both experience significant drops, but ${\breve{S_1}}$ has a slight recovery, showing resistance in the population against ${\breve{I_1}}$. The $\breve{R}$ compartment’s steady rise and plateau suggest that recovery is the primary outcome for infected individuals, which is a positive sign in terms of public health.

#### Real data

**Figure 3:**
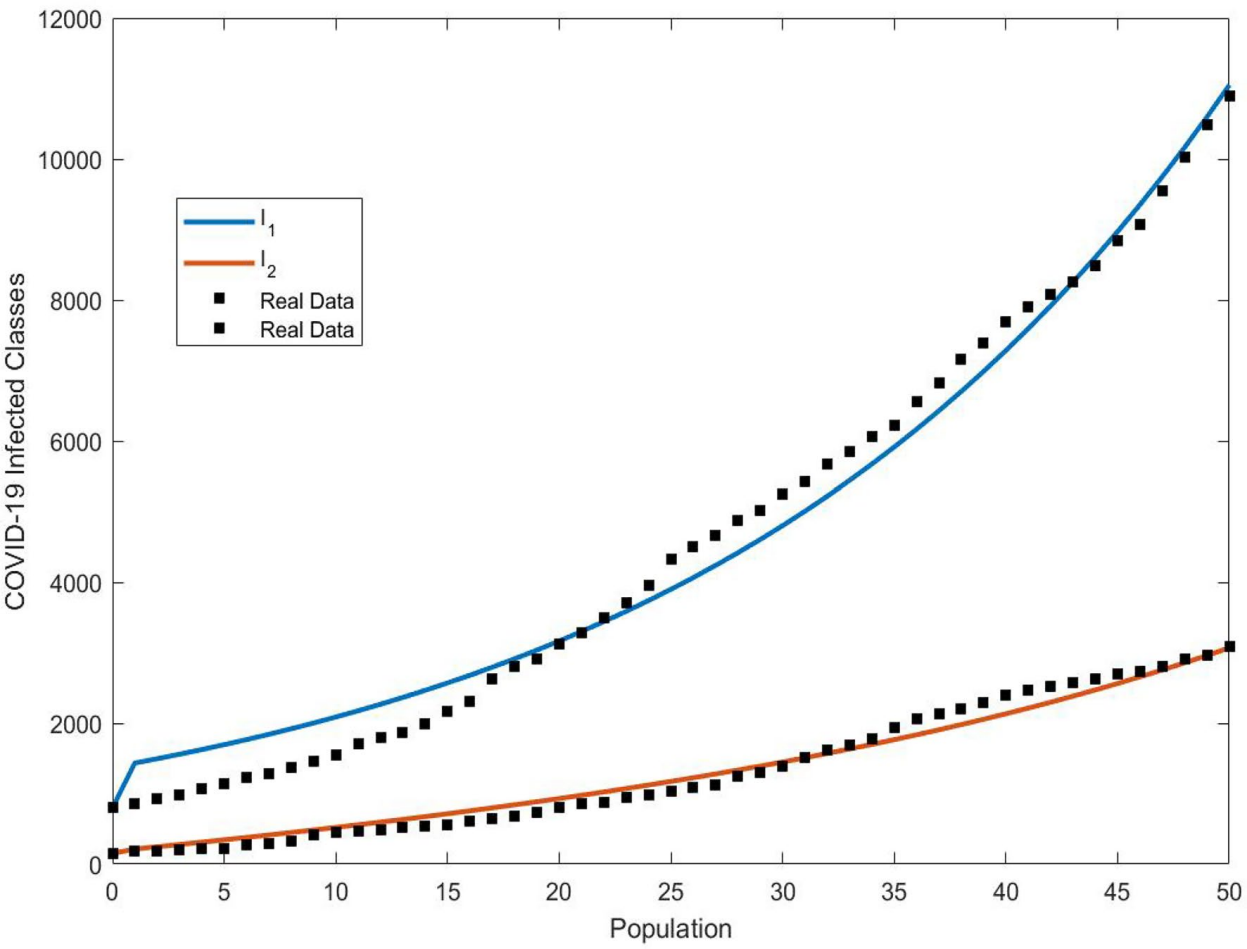
Real data analysis of COVID-19.

In Fig. 3, real data has been utilized to calibrate the model parameters for the alpha and beta variants of the SARS-CoV-2 virus given by Table 2, drawing on extensive datasets described in contemporary literature^11,35,39–43^. We have employed the nonlinear least square method for the estimation process. The Ordinary Least Square (OLS) solution was implemented to minimize the error terms as delineated in Eq. (7), and the related relative error was employed to assess the goodness of fit. Figure 3 illustrates the infected population as predicted by our proposed system with the real represented by stars.

7 $$\begin{aligned} \min \bigg \{\frac{\sum _{i=1}^{n}\left( I_{i}-{\hat{I}}_{i}\right) ^{2}}{\sum _{i=1}^{n} I_{i}^{2}}\bigg \}. \end{aligned}$$

Here the notion $I_{i}$ is the reported cumulative infected cases and ${\hat{I}}_{i}$ is the cumulative infected cases obtained from simulating the model.

**Table 2:** Adjusted parametric values.

Parameter	Value	Source
$\breve{S}_{1_0}$	0.475	Estimated
$\breve{S}_{2_0}$	0.475	Estimated
$\breve{I}_{1_0}$	0.01	Estimated
$\breve{I}_{2_0}$	0.03	Estimated
$\breve{R}_{0}$	0.0	^43^
$\beta _0$	0.6	^40^
$\alpha$	0.2	^40^
*r*	1.2	Estimated
*K*	1.2	Estimated
$\gamma$	0.08	Estimated
$\mu$	0.02	Estimated
$\delta$	0.01	^36,39^
$\sigma$	0.02	Estimated
$\rho$	0.01	^36,39^

### Phase plane plot

**Figure 4:**
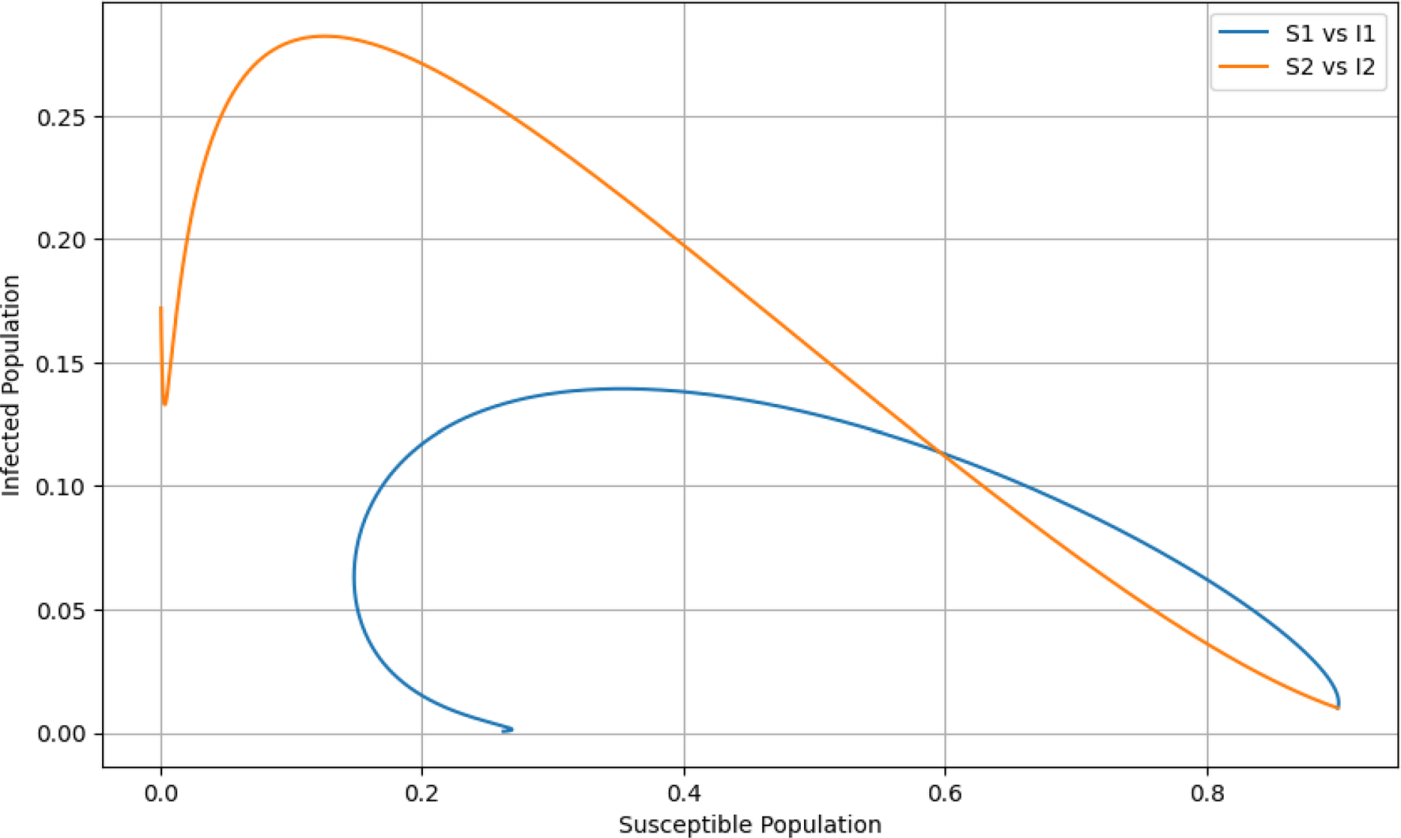
Phase plane plot.

The trajectories in Fig. 4 reveal the cyclical nature of the interactions between susceptibility and infection, illuminating the ebb and flow of the epidemic. As susceptibles are depleted, the number of new infections decreases, leading to a decline in the infected populations. While ${\breve{I_1}}$ have a cyclical pattern with ${\breve{S_1}}$, indicating periods of outbreak and control, ${\breve{I_2}}$ shows a more direct path to depletion of susceptibles, suggesting a more virulent or transmissible strain. This underlines the importance of strain-specific public health interventions.

### Time series with moving average

**Figure 5:**
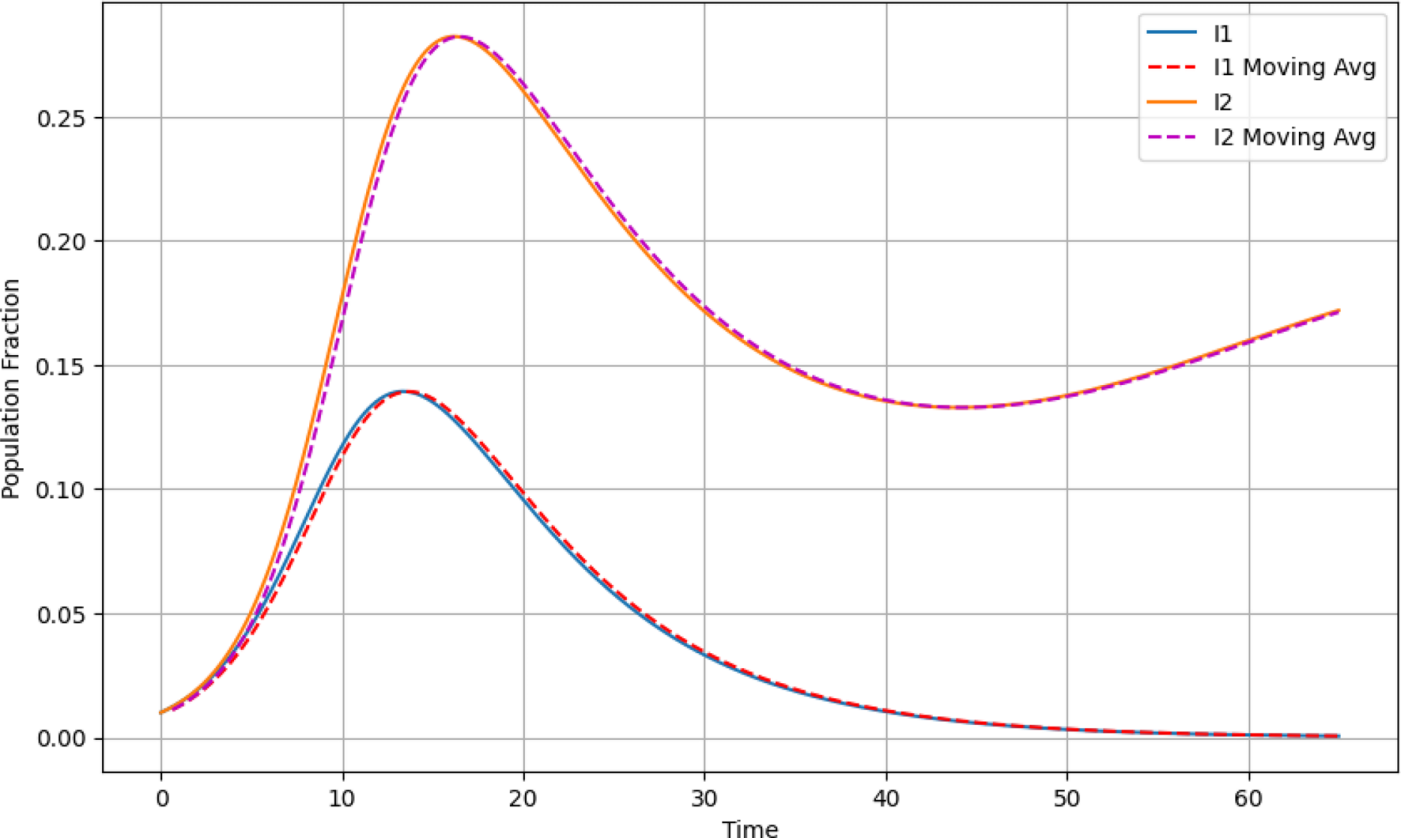
Time series with moving average.

While the raw data for ${\breve{I_1}}$ and ${\breve{I_2}}$ captures the actual number of infections, the moving average smoothens out short-term fluctuations, thereby highlighting broader trends. Both the actual infection data and the moving average exhibit a prominent peak, representing the height of the epidemic before a decline sets in. This underscores the transient nature of outbreaks and the eventual return to equilibrium as shown by Fig. 5.

### Quiver plot

**Figure 6:**
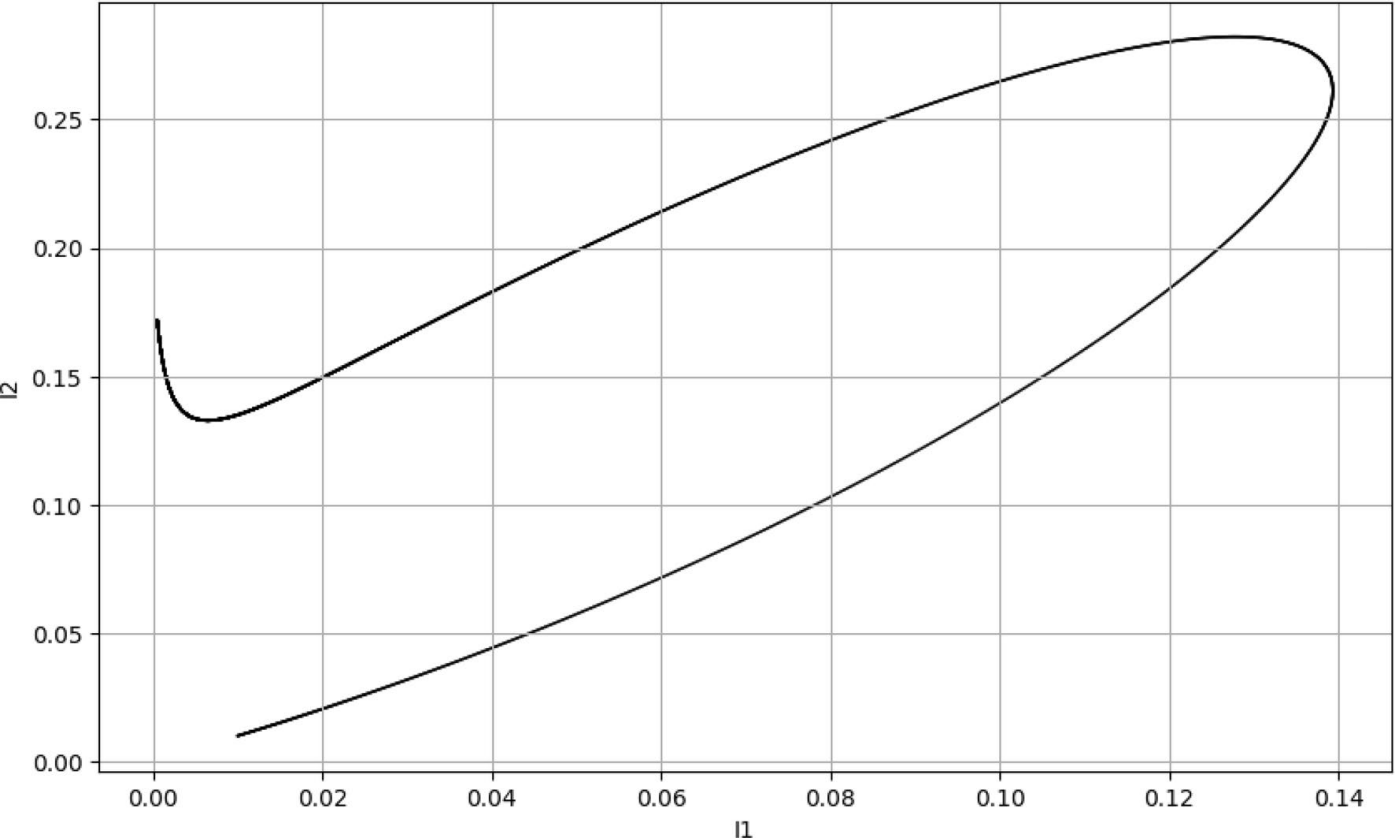
Quiver plot.

The quiver plot in Fig. 6 suggests complex interactions between the ${\breve{I_1}}$ and ${\breve{I_2}}$ strains. The arrows elucidate the direction and magnitude of change, providing a snapshot of the evolution in infection rates. The looping trajectory show oscillations in the infected populations, suggesting periodic outbreaks or potential for repeated waves of infections. This cyclic pattern arises from various factors, such as loss of immunity, seasonal variations, or reintroduction of infections.

### Population distribution

**Figure 7:**
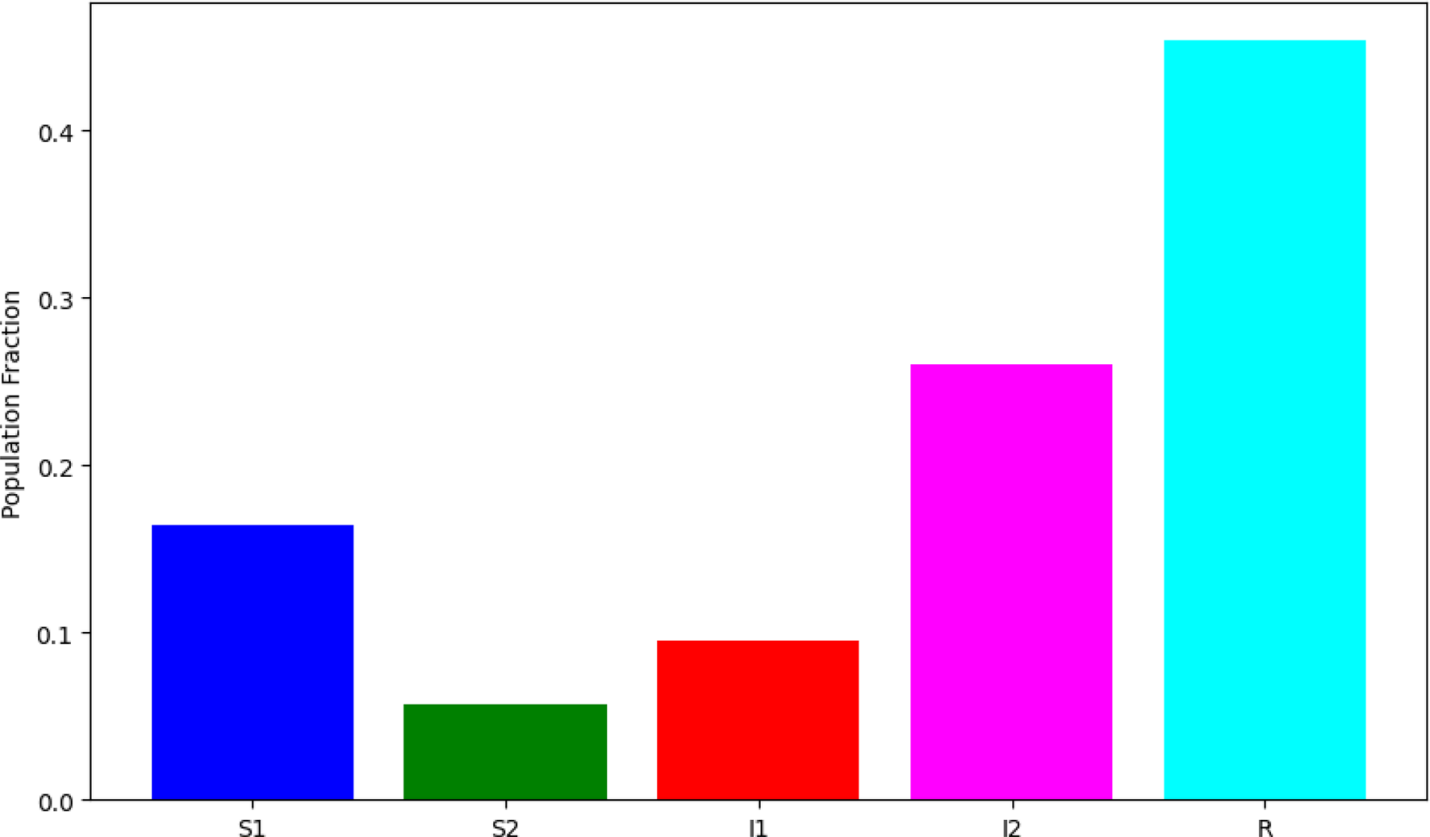
Population distribution at t = 100.

This bar graph gives an overview of the distribution of different populations at a specific time, $t = 100$ in Fig. 7. By this time, a significant portion of the initial susceptible population have transitioned to the recovered category. The number of infected individuals (${\breve{I_1}}$ and ${\breve{I_2}}$) is notably less than the susceptible and recovered groups, indicating that the epidemic is waning.

## Conclusion

The introduced epidemiological model represents a significant enhancement in the mathematical study of infectious diseases, especially those with multiple circulating strains such as SARS-CoV-2. Authenticated by real data analysis, the model’s equations provide a precise depiction of the disease dynamics, offering authenticity and bolstering its credibility within the scientific community.

The analysis of $\breve{I}_1$ and $\breve{I}_2$ through this model showcases a comprehensive mathematical characterization of the spread and potential control of multiple viral strains. The incorporation of empirical data not only underscores the model’s accuracy but also fortifies its predictive capacity regarding disease progression and potential extinction scenarios. The model’s robustness is affirmed through rigorous exploration of disease extinction conditions, laying a foundation for understanding the critical thresholds that govern the eradication of the disease.

The research by^10^ models COVID-19’s transmission with a focus on variant dynamics and vaccine impact, while^11^ assesses the influence of variants in France using optimization for parameter estimation. The study in^12^ introduces a fractional model for two COVID-19 strains, and^1^ explores the impact of multiple strains on pandemic trajectories and vaccine efficacy. These studies contribute valuable insights into pandemic modeling; however, our work extends these efforts by offering a more intricate examination of disease dynamics, specially;

- Integrates a wider range of epidemiological and evolutionary dynamics.
- Provides a deeper analytical approach including a comprehensive equilibrium analysis, and disease extinction conditions.
- Offers a more detailed exploration of both deterministic and stochastic scenarios.
- Utilizes advanced graphical techniques for a clearer understanding of disease progression.
- Addresses practical applications for real-world outbreaks, especially in the context of evolving viral strains impacting public health measures.

## Remarks and future recommendations

Our in-depth examination of the co-evolution model of host and human immune response highlights the intricate dynamics between infectious strains ${\breve{I_1}}$ and ${\breve{I_2}}$. The stark differences in the trajectories of these strains underscore the complex challenges posed in managing multi-strain infectious diseases. While our model offers a comprehensive understanding, the continually evolving nature of infectious diseases calls for adaptive strategies and persistent refinement in modeling approaches.

For future studies, it is recommended to incorporate factors like varying mutation rates, potential cross-immunity effects, and the impact of external interventions such as vaccination campaigns. Additionally, integrating real-world data can augment the model’s predictive accuracy. As global communities grapple with the multifaceted challenges posed by infectious diseases, models such as ours serve as foundational tools, and their continuous refinement remains pivotal for informed public health decision-making.

### Supplementary Information

Supplementary Information.

## Data Availability

All data generated or analysed during this study are included in this published article.
