# Supplementary material for: A stochastic approach for co-evolution process of virus and human immune system
Source: Sci Rep. 2024 May 6;14:10337. doi: 10.1038/s41598-024-60911-z (PMC11074307; doi:10.1038/s41598-024-60911-z)
Supplement: Supplementary file 1 — Supplementary Information. [file 41598_2024_60911_MOESM1_ESM.pdf]

12 Appendix

12.1 Equilibrium points

12.1.1 $E_3(0,1,0,0,0)$

Given the equilibrium $\mathfrak{E}_3(0,1,0,0,0)$ where $\breve{I_{1e}} = 0$, $\breve{I_{2e}} = 1$, $\breve{S_{1e}} = \breve{S_{2e}} = \breve{R_e} = 0$, we get,,

$$\mathcal{V}_{\mathfrak{E}_3} = \begin{pmatrix} -\beta_2 - \delta & 0 & \mu & \mu & \rho \\ -\beta_1 & 0 & 0 & 0 & 0 \\ 0 & 0 & -\gamma - \sigma - \delta & 0 & 0 \\ \beta_2 & 0 & 0 & -\gamma - \sigma - \delta & 0 \\ \gamma & \gamma & -\rho & -\rho & -\delta \end{pmatrix}.$$

The eigenvalues are, $-\beta_2 - \delta$, 0,$-\gamma - \sigma - \delta$,$-\gamma - \sigma - \delta$,$-\delta$.

Theorem 1. *The stability point $E_3(0,1,0,0,0)$ exhibits local asymptotic stability provided that $\beta_2 + \delta > 0$, $\gamma + \sigma + \delta > 0$, $\delta > 0$.*

12.1.2 $E_4(0,0,1,0,0)$

Given the variational matrix $\mathcal{V}$, we get,

$$\mathcal{V} = \begin{pmatrix} -\delta & -\beta_1 - \beta_2 & \mu - \beta_1 & \mu & \rho \\ 0 & -\delta & \beta_1 & 0 & 0 \\ 0 & \beta_1 & -\gamma - \sigma - \delta & 0 & 0 \\ 0 & 0 & 0 & -\gamma - \sigma - \delta & 0 \\ \gamma & \gamma & -\rho & -\rho & -\delta \end{pmatrix}.$$

The eigenvalues of this matrix are $-\delta$, $-\delta$, $-\gamma - \sigma - \delta$, $-\gamma - \sigma - \delta$, $-\delta$.

Theorem 2. *The stability point $E_4(0,0,1,0,0)$ exhibits local asymptotic stability provided that $\delta > 0$,$\gamma + \sigma + \delta > 0$.*

12.1.3 $E_5(0,0,0,1,0)$

For the equilibrium $\mathfrak{E}_5(0,0,0,1,0)$, where $\breve{I_{1e}} = 0$, $\breve{I_{2e}} = 0$, $\breve{S_{1e}} = 0$, $\breve{S_{2e}} = 1$, and $\breve{R_e} = 0$ is

$$\mathcal{V} = \begin{pmatrix} -\delta & -\beta_1 - \beta_2 & \mu & \mu - \beta_2 & \rho \\ 0 & -\delta & 0 & \beta_2 & 0 \\ 0 & 0 & -\gamma - \sigma - \delta & 0 & 0 \\ 0 & \beta_2 & 0 & -\gamma - \sigma - \delta & 0 \\ \gamma & \gamma & -\rho & -\rho & -\delta \end{pmatrix}.$$

The eigenvalues of this matrix are $-\delta$, $-\delta$, $-\gamma - \sigma - \delta$,$-\gamma - \sigma - \delta$, $-\delta$.

Theorem 3. *The stability point $E_1(0,0,0,1,0)$ exhibits local asymptotic stability provided that $\delta > 0$, $\gamma + \sigma + \delta > 0$.*

12.1.4 $E_6(0,0,0,0,1)$

Given the variational matrix

$$\mathcal{V} = \begin{pmatrix} -\delta & 0 & \mu & \mu & \rho \\ 0 & 0 & 0 & 0 & 0 \\ 0 & 0 & -\gamma-\sigma-\delta & 0 & 0 \\ 0 & 0 & 0 & -\gamma-\sigma-\delta & 0 \\ \gamma & \gamma & -\rho & -\rho & -\delta \end{pmatrix}.$$

The eigenvalues of this matrix are $-\delta$, 0, $-\gamma-\sigma-\delta$,$-\gamma-\sigma-\delta$,$-\delta$.

Theorem 4. *The stability point $E_1(0,0,0,0,1)$ exhibits local asymptotic stability provided that $\delta > 0$, $\gamma+\sigma+\delta > 0$.*

12.1.5 $E_7(1,1,0,0,0)$

For the equilibrium point $\mathfrak{E}_7(1,1,0,0,0)$ where $\breve{I_{1e}} = 1$, $\breve{I_{2e}} = 1$, $\breve{S_{1e}} = 0$, $\breve{S_{2e}} = 0$, and $\breve{R_e} = 0$, we have the variational matrix,

$$\mathcal{V} = \begin{pmatrix} -\beta_1-\beta_2-\delta & 0 & \mu & \mu & \rho \\ -\beta_2-\beta_1 & 0 & 0 & 0 & 0 \\ 0 & \beta_1 & -\gamma-\sigma-\delta & 0 & 0 \\ 0 & \beta_2 & 0 & -\gamma-\sigma-\delta & 0 \\ \gamma & \gamma & -\rho & -\rho & -delta \end{pmatrix}.$$

The eigenvalues of this matrix are the solutions to the determinant of $\mathcal{V} - \lambda I = 0$, where I is the identity matrix. $[-1.43069623 + 0.j - 0.21201624 + 0.22919424j - 0.21201624 - 0.22919424j, -0.4452713 + 0.j - 0.8 + 0.j]$. Given the negative real parts of all eigenvalues, the stability point $E_7(1,1,0,0,0)$ exhibits local asymptotic stability.

12.1.6 $E_8 1,1,1,0,0$

Substitute the equilibrium values into the matrix

$$\mathcal{V} = \begin{pmatrix} -\beta_1-\beta_2-\delta & -\beta_1-\beta_2 & \mu-\beta_1 & \mu-\beta_2 & \rho \\ -\beta_2-\beta_1 & -\beta_2 & \beta_1 & \beta_2 & 0 \\ \beta_1 & \beta_1 & -\gamma-\sigma-\delta & 0 & 0 \\ \beta_2 & \beta_2 & 0 & -\gamma-\sigma-\delta & 0 \\ \gamma & \gamma & -\rho & -\rho & -\delta \end{pmatrix}.$$

Theorem 5. *The stability point $E_8(1,1,1,0,0)$ exhibits local asymptotic stability provided that*

1. $\beta_1+\beta_2+\delta > k_1(\beta_1+\beta_2)$ *where* $k_1 > 1$.
2. $\mu+\beta_1-\beta_2\breve{S_{1e}} < k_2(\beta_1-\beta_2\breve{S_{1e}})$ *where* $0 < k_2 < 1$.
3. $\gamma+\sigma+\delta > k_3(\gamma+\sigma)$ *where* $k_3 > 1$.
4. $\mu+\beta_2 > k_4\beta_2$ *where* $k_4 > 1$.
5. $\gamma+\rho+\delta > k_5(\gamma+\rho)$ *where* $k_5 > 1$.

12.1.7 $E_9 1, 1, 1, 1, 0$

Substituting the equilibrium values, we get:

$$V = \begin{bmatrix} -\beta_1 - \beta_2 - \delta & -\beta_2 & \beta_2 & 0 & 0 \\ -\beta_1 - \beta_2 & -\beta_1 - \delta & 0 & \beta_1 & 0 \\ \beta_1 & 0 & -\gamma - \sigma - \delta & -\beta_1 & \gamma \\ 0 & \beta_2 & \beta_2 & -\mu - \beta_2 - \delta & \gamma \\ 0 & 0 & \sigma & \rho & -\gamma - \rho - \delta \end{bmatrix}.$$

[-1.63103353+0.j 0.04932644+0.j -0.84360343+0.24368374j, -0.84360343-0.24368374j -0.38108607+0.j] The stability point $E_9(1, 1, 1, 1, 0)$ exhibits local asymptotic stability provided that

1. $\beta_1 + \beta_2 + \delta > k_1(\beta_1 + \beta_2)$ where $k_1 > 1.63103353$.
2. $\mu + \beta_1 - \beta_2 \breve{S_{1e}} < k_2(\beta_1 - \beta_2 \breve{S_{1e}})$ where $0 < k_2 < 0.04932644$.
3. $\gamma + \sigma + \delta > k_3(\gamma + \sigma)$ where $k_3 > 1.08728717$ (considering the largest magnitude between third and fourth eigenvalues).
4. $\mu + \beta_2 > k_4\beta_2$ where $k_4 > 1.38108607$.
5. $\gamma + \rho + \delta > k_5(\gamma + \rho)$ where $k_5 > 1.38108607$.

12.2 Proof of theorem 5

Proof. Consider the function $V(\breve{I}_1, \breve{I}_2) = \breve{I}_1 + \breve{I}_2$. The time derivative of V along the trajectories of the system is:

$$\frac{dV}{dt} = \frac{dI_1}{dt} + \frac{dI_2}{dt}.$$

Using the system of equations, we can show that $\frac{dV}{dt} \leq 0$ for all $\breve{I}_1, \breve{I}_2 > 0$.

$$\frac{dI_1}{dt} = \beta_1 \breve{S}_2 \breve{I}_1 - \gamma \breve{I}_1 - \sigma \breve{I}_1 - \delta \breve{I}_1.$$

$$\frac{dI_2}{dt} = \beta_2 \breve{S}_1 \breve{I}_2 - \gamma \breve{I}_2 - \sigma \breve{I}_2 - \delta \breve{I}_2.$$

Substituting in the given equations:

$$\frac{dV}{dt} = \beta_1 \breve{S}_2 \breve{I}_1 + \beta_2 \breve{S}_1 \breve{I}_2 - \gamma(\breve{I}_1 + \breve{I}_2) - \sigma(\breve{I}_1 + \breve{I}_2) - \delta(\breve{I}_1 + \breve{I}_2).$$

Now, let's analyze the terms:

1. The terms $\beta_1 \breve{S}_2 \breve{I}_1$ and $\beta_2 \breve{S}_1 \breve{I}_2$ represent the new infections. As the number of infected individuals increases, the number of susceptible individuals (either $\breve{S}_1$ or $\breve{S}_2$) decreases. This means that these terms will decrease as $\breve{I}_1$ and $\breve{I}_2$ increase.

2. The terms $-\gamma(\breve{I}_1 + \breve{I}_2)$, $-\sigma(\breve{I}_1 + \breve{I}_2)$, and $-\delta(\breve{I}_1 + \breve{I}_2)$ are all negative and increase in magnitude as $\breve{I}_1$ and $\breve{I}_2$ increase.

As $\breve{I}_1$ and $\breve{I}_2$ increase, the positive terms in $\frac{dV}{dt}$ decrease, while the negative terms increase in magnitude. This ensures that $\frac{dV}{dt}$ is non-positive for all $\breve{I}_1, \breve{I}_2 > 0$, i.e., $\frac{dV}{dt} \leq 0$.

Thus, we've shown that $\frac{dV}{dt} \leq 0$ for all $\breve{I}_1, \breve{I}_2 > 0$. □

12.3 Proof of theorem 6

Proof. Consider $(\breve{S}_1(\mathrm{t}), \breve{S}_2(\mathrm{t}), \breve{I}_1(\mathrm{t}), \breve{I}_2(\mathrm{t}), \breve{R}(\mathrm{t}))$ to be any solution of the system.

Combining the differential equations for $\breve{S}_1$ and $\breve{S}_2$, we get:

$$\frac{d\breve{S}_1}{dt} + \frac{d\breve{S}_2}{dt} \leq \mu + \rho\breve{R} - \delta(\breve{S}_1 + \breve{S}_2). \tag{1}$$

From the above inequality, we deduce that

$$\lim_{\mathrm{t}\to\infty} \sup(\breve{S}_1(\mathrm{t}) + \breve{S}_2(\mathrm{t})) \leq \frac{\mu + \rho\breve{R}}{\delta}. \tag{2}$$

Let,

$$W(\mathrm{t}) = \breve{S}_1(\mathrm{t}) + \breve{S}_2(\mathrm{t}) + \breve{I}_1(\mathrm{t}) + \breve{I}_2(\mathrm{t}) + \breve{R}(\mathrm{t}). \tag{3}$$

Then, summing up all the differential equations, we have,

$$\frac{dW}{dt} \leq \mu + \rho R - \delta W, \tag{4}$$

which can be simplified as

$$\frac{dW}{dt} + \delta W \leq \mu + \rho\breve{R}. \tag{5}$$

From differential inequalities theory,

$$0 \leq W(\mathrm{t}) \leq \frac{\mu + \rho\breve{R}}{\delta} + \frac{W(0)}{e^{\delta\mathrm{t}}}. \tag{6}$$

Taking the limit as $\mathrm{t} \to \infty$,

$$0 \leq W \leq \frac{\mu + \rho\breve{R}}{\delta}. \tag{7}$$

Therefore, for any $\epsilon > 0$,

$$C = \{(\breve{S}_1, \breve{S}_2, \breve{I}_1, \breve{I}_2, \breve{R}) : 0 \leq W \leq \frac{\mu + \rho\breve{R}}{\delta} + \epsilon\}. \tag{8}$$

□

12.4 Proof of theorem 7

Let $l^* \in \mathbb{N}^*$ s.t. $Z(0) \in \left[\frac{1}{l^*}, \frac{\nu}{\epsilon}\right]$. For $n \geq l^*$, we denote:

$$\sigma_n = \inf\left\{ \mathrm{t} > 0 \middle| Z(\mathrm{t}) \in \boldsymbol{\Xi} \text{ and } Z(\mathrm{t}) \notin \left[\frac{1}{n}, \frac{\nu}{\epsilon}\right]^5 \right\},$$

here σ_n representing the stopping time with $\sigma = \inf\{0 < \mathrm{t} : Z(\mathrm{t}) \notin \boldsymbol{\Xi}\}$.

The task remains to show $\mathcal{P}\{\mathrm{t} > \sigma\} = 0$ for every $\mathrm{t} > 0$.

$\sigma_n < \sigma$ implies $\mathcal{P}\{\sigma_n \leq \mathrm{t}\} \geq \mathcal{P}\{\sigma < \mathrm{t}\}$.

If $\lim_{n\to\infty} \sup \mathcal{P}\{\sigma_n < \mathrm{t}\} = 0$, then our assertion holds. For the outset, let's take Θ_1: $\mathbb{R}_+^{4*} \to \mathbb{R}_+$ as a Lyapunov function.

$$\Im_1(\breve{S}_1(\mathrm{t}), \breve{S}_2(\mathrm{t}), \breve{I}_1(\mathrm{t}), \breve{I}_2(\mathrm{t}), R(\mathrm{t}) = \breve{S}_1^{\,2}(\mathrm{t}) + \breve{S}_2^{\,2}(\mathrm{t}) + \breve{I}_1^{\,2}(\mathrm{t}) + \breve{I}_2^{\,2}(\mathrm{t}) + R^2(\mathrm{t}) \tag{9}$$

Using Ito's formula, whenever $0 < \mathrm{t}$ and $\varrho \in [0, \mathrm{t} \wedge \sigma_n]$, we get

$$\begin{aligned} &d\Im_1(\breve{S}_1(\mathrm{t}), \breve{S}_2(\mathrm{t}), \breve{I}_1(\mathrm{t}), \breve{I}_2(\mathrm{t}), R(\mathrm{t}) = \mathbf{H}\Im_1 dt - \varrho_1 \breve{S}_1^{\,2} d\mathfrak{D}_1(\mathrm{t}) - \varrho_2 \breve{S}_2^{\,2} d\mathfrak{D}_2(\mathrm{t}) \\ &- \varrho_3 \breve{I}_1^{\,2} d\mathfrak{D}_3(\mathrm{t}) - \varrho_4 \breve{I}_2^{\,2} d\mathfrak{D}_4(\mathrm{t}) - \varrho_5 R^2 d\mathfrak{D}_5(\mathrm{t}), \end{aligned}$$

where

$$\begin{aligned} \mathbf{H}\Im_1 = &- \mu \breve{S}_1^{\,2} - \beta_1 \breve{S}_1^{\,3} \breve{I}_1 - \beta_2 \breve{S}_1^{\,3} \breve{I}_2 + \rho R \breve{S}_1^{\,2} - \delta \breve{S}_1^{\,2} \\ &- \beta_2 \breve{S}_2^{\,3} \breve{I}_2 - \beta_1 S_2^2 \breve{I}_1 - \delta \breve{S}_2^{\,3} + \beta_1 \breve{S}_2 \breve{I}_1^{\,2} \\ &- \gamma \breve{I}_1^{\,3} - \sigma \breve{I}_1^{\,3} - \delta \breve{I}_1^{\,3} - \beta_2 \breve{S}_1 \breve{I}_2^{\,3} - \gamma \breve{I}_2^{\,3} \\ &- \sigma \breve{I}_2^{\,3} - \delta \breve{I}_2^{\,3} - \gamma \breve{I}_1 \breve{R}^2 + \gamma \breve{I}_2 \breve{R}^2 - \rho \breve{R}^3 - \delta \breve{R}^3 \\ &+ \varrho_1^{\,2} \breve{S}_1^{\,2} + \varrho_2^{\,2} \breve{S}_2^{\,2} + \varrho_3^{\,2} \breve{I}_1^{\,2} + \varrho_4^{\,2} \breve{I}_2^{\,2} + \varrho_5^{\,2} \breve{R}^2. \end{aligned}$$

then

$$\mathbf{H}\Im_1 \leq \rho \breve{R} \breve{S}_1^{\,2} + \beta_1 \breve{S}_2 \breve{I}_1^{\,2} + \gamma \breve{I}_2 R^2 + \varrho_1^{\,2} \breve{S}_1^{\,2} + \varrho_2^{\,2} \breve{S}_2^{\,2} + \varrho_3^{\,2} \breve{I}_1^{\,2} + \varrho_4^{\,2} \breve{I}_2^{\,2} + \varrho_5^{\,2} \breve{R}^2$$

then we have,

$$\begin{aligned} d\Im_1 \leq\ & \rho + \beta_1 + \gamma + \varrho_1^2 + \varrho_2^2 + \varrho_3^2 + \varrho_4^2 \\ &- \frac{\varrho_1}{\mathfrak{s}} d\mathfrak{D}_1(\mathrm{t}) - \frac{\varrho_2}{\mathfrak{i}_1} d\mathfrak{D}_2(\mathrm{t}) - \frac{\varrho_3}{\mathfrak{i}_2} d\mathfrak{D}_3(\mathrm{t}) - \frac{\varrho_4}{\mathfrak{i}_3} d\mathfrak{D}_4(\mathrm{t}) \\ \leq\ & \theta dt - \varrho_1 \breve{S}_1 d\mathfrak{D}_1(\mathrm{t}) - \varrho_2 \breve{S}_2 d\mathfrak{D}_2(\mathrm{t}) - \varrho_3 \breve{I}_1 d\mathfrak{D}_3(\mathrm{t}) \\ &- \varrho_4 \breve{I}_2 d\mathfrak{D}_4(\mathrm{t}) - \varrho_5 \breve{R} d\mathfrak{D}_5(\mathrm{t}). \end{aligned} \tag{10}$$

$$\theta = \left[\rho + \beta_1 + \gamma + \varrho_1^2 + \varrho_2^2 + \varrho_3^2 + \varrho_4^2\right].$$

By utilizing the principles of expectation and the Fubbini theorem, integrating the aforementioned equation yields,

$$\mathcal{F}(\Im_1(Z(\varrho)) \leq \Im_1(Z_0) + \theta\varrho.$$

Invoking the results from Gronvall, for every $\varrho \in [0, \mathrm{t} \wedge \sigma_n]$

$$\mathcal{F}(\Im_1(Z(\varrho)) \leq \Im_1(Z_0)e^{\theta\varrho},$$

we deduce,

$$\mathcal{F}[\Im_1(Z(\mathrm{t} \wedge \tau_n)] \leq \Im_1(Z_0) + \theta\mathrm{t}. \tag{11}$$

Given that $\Im_1(Z(\mathrm{t} \wedge \sigma_n)) > 0$ and $Z(\sigma_n) \leq \frac{1}{n}$, it follows,

$$\begin{aligned} \mathcal{F}(\Im_1(Z(\mathrm{t} \wedge \sigma_n)) &\geq \mathcal{F}(\Im_1(m(\sigma_n))\mathbf{1}_{\{\sigma_n < \mathrm{t}\}}), \\ &\geq n\mathrm{B}(\sigma_n < \mathrm{t}). \end{aligned} \tag{12}$$

From the implications of equations (11) and (12), for all $\mathrm{t} \geq 0$,

$$\mathrm{B}(\sigma_n < \mathrm{t}) \leq \frac{\Im(Z_0) + \theta\mathrm{t}}{n}.$$

Consequently,

$$\lim_{\mathrm{t} \to \infty} \sup \mathrm{B}(\sigma_n < \mathrm{t}) = 0.$$

12.5 Proof of theorem 8

Let's assume that for any initial condition $\Big(\breve{S}_1(0), \tilde{S}_2(0), \tilde{I}_1(0), \tilde{I}_2(0), \breve{R}(0)\Big) \in \Xi$, there exists a unique and positive local solution of the form $\Big(S1(\mathrm{t}), \breve{S}_2(\mathrm{t}), \breve{I}_1(\mathrm{t}), \breve{I}_2(\mathrm{t}), R(\mathrm{t})\Big)$ over the interval $[0, \sigma_e)$. The term σ_e denotes the time of explosion. We aim to prove $\sigma_e = \infty$ almost surely, implying the solution is global. Furthermore, let's define σ_{l^*} as our chosen stopping time It is given that the infimum of ϕ is infinite. As j tends towards infinity, it can be observed that σ_{l^*} exhibits an increasing behavior. If we define σ_∞ as $\lim\limits_{j \to \infty} \sigma_{l^*}$, it follows that $\sigma_e \geq \sigma_\infty$ with high probability. Our goal is to demonstrate that $\sigma_\infty = \infty$ almost surely.

$$\begin{aligned} \sigma_{l^*} = &\inf \mathrm{t} \in (0, \sigma_e) : \breve{S}_1(\mathrm{t}) \notin (\frac{1}{l^*}, j) \text{ or } \breve{S}_2(\mathrm{t}) \notin (\frac{1}{l^*}, j) \\ &\text{or } \breve{I}_1(\mathrm{t}) \notin (\frac{1}{l^*}, j) \text{ or } \breve{I}_2(\mathrm{t}) \notin (\frac{1}{l^*}, j) \text{ or } \breve{R}(\mathrm{t}) \notin (\frac{1}{l^*}, j). \end{aligned} \tag{13}$$

Assuming, for the sake of contradiction, that $\sigma_\infty = \infty$ isn't always true, we would then have some non-negative Q and a value of ε within [0,1) such that the probability $\mathrm{D}(\sigma_\infty < Q) > \varepsilon$. This implies:

$$\exists(l_1^* \in \mathrm{N})(\forall l^* \geq l^*1)\mathrm{D}(\sigma l^* \leq Q) \geq \varepsilon. \tag{14}$$

Let's further introduce the function $\Im_2$, a twice continuously differentiable function mapping from R_4^+ to R^+, given by

$$\Im_2(\breve{S}_1(\mathrm{t}), \breve{S}_2(\mathrm{t}), \breve{I}_1(\mathrm{t}), \breve{I}_2(\mathrm{t}), \breve{R}(\mathrm{t}), = \left(\breve{S}_1 - 1 - \ln \breve{S}_1\right) + \left(\breve{S}_2 - 1 - \ln \breve{S}_2\right) + \left(\breve{I}_1 - 1 - \ln \breve{I}_1\right) + \left(\breve{I}_2 - 1 - \ln \breve{I}_2\right).$$

Applying Ito's formula, we derive

$$\begin{aligned} d\Im_2 = \mathbf{H}\Im_2 & \left(\breve{S}_1, \breve{S}_2, \breve{I}_1, \breve{I}_2\right) dt \\ & + \left(\breve{S}_1 - 1\right) \varrho_1 d\mathfrak{D}_1(\mathrm{t}) + (S_2 - 1)\, \varrho_2 d\mathfrak{D}_2(\mathrm{t}) \\ & + \left(\breve{I}_1 - 1\right) \varrho_3 d\mathfrak{D}_3(\mathrm{t}) + \left(\breve{I}_2 - 1\right) \varrho_4 d\mathfrak{D}_4(\mathrm{t}) \\ & + \left(\breve{R} - 1\right) \varrho_5 d\mathfrak{D}_5(\mathrm{t}), \end{aligned}$$

Where the expression for $\mathbf{H}\Im_2$ is,

$$\begin{aligned} \mathbf{H}\Im_2 = & \ \mu - \beta_1 \breve{S}_1 \breve{I}_1 - \beta_2 \breve{S}_1 \breve{I}_2 + \rho \breve{R} - \delta \breve{S}_1 - (\mu - \beta_1 \breve{I}_1 - \beta_2 \breve{I}_2 + \rho \breve{R} - \delta) \\ & - \beta_2 \breve{S}_2 \breve{I}_2 - \beta_1 \breve{S}_2 \breve{I}_1 - \delta \breve{S}_2 - (-\beta_2 \breve{I}_2 - \beta_1 \breve{I}_1 - \delta) + \beta_1 \breve{S}_2 - \gamma - \sigma - \delta \\ & + \beta_2 \breve{S}_1 \breve{I}_2 - \gamma \breve{I}_2 - \sigma \breve{I}_2 - \delta \breve{I}_2 (\beta_2 \breve{S}_1 - \gamma - \sigma - \delta) + \gamma \breve{I}_1 + \gamma \breve{I}_2 - \rho \breve{R} - \delta \breve{R} + \\ & (\gamma \breve{I}_1 + \gamma \breve{I}_2 - \rho - \delta) + \frac{1}{2}{\varrho_1}^2 dt + \frac{1}{2}{\varrho_2}^2 dt + \frac{1}{2}{\varrho_3}^2 dt + \frac{1}{2}{\varrho_4}^2 dt + \frac{1}{2}{\varrho_5}^2 dt. \end{aligned}$$

This leads to the inequality

$$\begin{aligned} \mathbf{H}\Im_2 \leq & \ \mu + \rho \breve{R} - \delta \breve{S}_1 + \beta_1 \breve{I}_1 + \beta_2 \breve{I}_2 + \delta) \\ & + (\beta_2 \breve{I}_2 + \beta_1 \breve{I}_1 + \delta) + \beta_1 \breve{S}_2 + \beta_2 \breve{S}_1 \breve{I}_2 + \gamma + \sigma + \delta + \gamma \breve{I}_1 + \gamma \breve{I}_2 + (\gamma \breve{I}_1 + \gamma \breve{I}_2) + \\ & \frac{1}{2}{\varrho_1}^2 dt + \frac{1}{2}{\varrho_2}^2 dt + \frac{1}{2}{\varrho_3}^2 dt + \frac{1}{2}{\varrho_4}^2 dt + \frac{1}{2}{\varrho_5}^2 dt = \mathcal{J} \end{aligned}$$

Integration over the given interval yields

$$\begin{aligned} d\Im_2 \leq & \ \mathcal{J} + \left(\breve{S}_1 - 1\right) \varrho_1 d\mathfrak{D}_1(\mathrm{t}) + \left(\breve{S}_2 - 1\right) \varrho_2 d\mathfrak{D}_2(\mathrm{t}) + \left(\breve{I}_1 - 1\right) \varrho_3 d\mathfrak{D}_3(\mathrm{t}) + \\ & \left(\tilde{I}_2 - 1\right) \varrho_4 d\mathfrak{D}_4(\mathrm{t}) + \left(\breve{R} - 1\right) \varrho_5 d\mathfrak{D}_5(\mathrm{t}). \end{aligned} \tag{15}$$

$$\mathfrak{E}\Im_2\left(\breve{S}_1\left(Q\wedge\sigma_{l^*}\right),\breve{S}_2\left(T\wedge\sigma_{l^*}\right),\breve{I}_1\left(Q\wedge\sigma_{l^*}\right),\breve{I}_2\left(Q\wedge\sigma_{l^*}\right),\breve{R}\left(Q\wedge\sigma_{l^*}\right)\right)$$

$$\leq \Im_2\left(\breve{S}_1(0),\breve{S}_2(0),\breve{I}_1(0),\breve{I}_2(0),\breve{R}(0)\right)+\mathcal{J}\mathbb{E}\left(Q\wedge\sigma_{l^*}\right),$$

$$\leq \Im_2\left(\breve{S}_1(0),\breve{S}_2(0),\breve{I}_1(0),\breve{I}_2(0),\breve{R}(0)\right)+\mathcal{J}Q.$$

Considering the set Ω_{l^*} where $l_1^* \leq j$, and from the relation in (14), we can infer that,

$$\begin{aligned}&\Im_2\left(\breve{S}_1(0),\breve{S}_2(0),\breve{I}_1(0),\breve{I}_2(0),\breve{R}(0)\right)+\mathcal{J}Q\\&\geq \mathbb{E}\left(1_{\Omega_{l^*}}\Im_2\left(\breve{S}_1\left(\sigma_{l^*},\omega\right),\breve{S}_2\left(\tau_{j,}\omega\right),\breve{I}_1\left(\sigma_{l^*},\omega\right),\breve{I}_2\left(\sigma_{l^*},\omega\right),\breve{R}\left(\sigma_{l^*},\omega\right)\right)\right),\\&\geq \varepsilon[l^*-1-\ln l^*]\wedge\left[\frac{1}{l^*}-1+\ln l^*\right].\end{aligned}$$

Upon letting l^* approach infinity, we encounter the contradiction, $\infty > \Im_2\left(\breve{S}_1(0),\breve{S}_2(0),\breve{I}_1(0),\breve{I}_2(0),\breve{R}(0)\right)+\mathcal{J}Q=\infty$ a.s. This inconsistency with our initial assumption proves our desired result.

12.6 Proof of theorem 9

Let $\Im_3 = \ln\breve{I}_1(\mathrm{t})$.

$$\begin{aligned}d\ln\breve{I}_1(\mathrm{t}) &= \frac{1}{\breve{I}_1}dI_1-\frac{1}{2}{\varrho_2}^2dt,\\&=\left[\beta_1S_2-\gamma-\sigma-\delta-\frac{1}{2}{\varrho_2}^2\right]dt+\varrho_2 d\mathfrak{D}_2(\mathrm{t}),\\&\leq\left[\beta_1S_2-\delta-\frac{1}{2}{\varrho_2}^2\right]dt+\varrho_2 d\mathfrak{D}_2(\mathrm{t}).\end{aligned} \tag{16}$$

Integrating (16) gives

$$\begin{aligned}\ln\breve{I}_1(\mathrm{t}) &\leq \beta_1\int_0^{\mathrm{t}}\breve{S}_1(r)dr-\delta(\mathrm{t})-\frac{1}{2}{\varrho_2}^2\mathrm{t}+\varrho_2\mathfrak{D}_2(\mathrm{t})-\varrho_2\mathfrak{D}_2(0)+\ln\breve{I}_1(0),\\\beta_1 &\leq \int_0^{\mathrm{t}}\breve{S}_1(r)dr-\delta(\mathrm{t})-\frac{1}{2}{\varrho_2}^2\mathrm{t}+P_1(\mathrm{t}),\end{aligned} \tag{17}$$

where $P_1(\mathrm{t}) = \varrho_2\mathfrak{D}_2(\mathrm{t}) - \varrho_2\mathfrak{D}_2(0) + \ln \breve{I}_1(0)$. Dividing (17) by t, we have

$$\frac{\ln \breve{I}_1(\mathrm{t})}{\mathrm{t}} \le \beta_1\langle \breve{S}_1\rangle - \delta - \frac{1}{2}{\varrho_2}^2 + \frac{P_1(\mathrm{t})}{\mathrm{t}}. \tag{18}$$

Integrating the model, we can write

$$\begin{cases} \frac{\breve{S}_1(\mathrm{t})-\breve{S}_1(0)}{\mathrm{t}} & = \mu - \beta_1\int_0^{\mathrm{t}} \breve{S}_1\breve{I}_1 - \beta_2\int_0^{\mathrm{t}} \breve{S}_1\breve{I}_2 + \rho\langle R\rangle - \delta\langle \breve{S}_1\rangle + \int_0^{\mathrm{t}} \breve{S}_1(r)d\mathfrak{D}_1(r), \\ \frac{\breve{S}_2(\mathrm{t})-\breve{S}_2(0)}{\mathrm{t}} & = -\beta_2\int_0^{\mathrm{t}} S_2\breve{I}_2 - \beta_1\int_0^{\mathrm{t}} S_2\breve{I}_1 - \delta\langle S_2\rangle + \frac{\varrho_2}{\mathrm{t}}\int_0^{\mathrm{t}} \breve{S}_2(r)d\mathfrak{D}_2(r), \\ \frac{\breve{I}_1(\mathrm{t})-\breve{I}_1(0)}{\mathrm{t}} & = \beta_1\int_0^{\mathrm{t}} S_2\breve{I}_1 - \gamma\langle \breve{I}_1\rangle - \sigma\langle \breve{I}_1\rangle - \delta\langle \breve{I}_1\rangle + \frac{\varrho_3}{\mathrm{t}}\int_0^{\mathrm{t}} \breve{I}_1(r)d\mathfrak{D}_3(r), \\ \frac{\breve{I}_2(\mathrm{t})-\breve{I}_2(0)}{\mathrm{t}} & = \beta_2\int_0^{\mathrm{t}} \breve{S}_1\breve{I}_2 - \gamma\langle \breve{I}_2\rangle - \sigma\langle \breve{I}_2\rangle - \delta\langle \breve{I}_2\rangle + \frac{\varrho_4}{\mathrm{t}}\int_0^{\mathrm{t}} \breve{I}_2(r)d\mathfrak{D}_4(r), \\ \frac{\breve{R}(\mathrm{t})-\breve{R}(0)}{\mathrm{t}} & = \gamma\langle \breve{I}_1\rangle + \gamma\langle \breve{I}_2\rangle - \rho\langle \breve{R}\rangle - \delta\langle \breve{R}\rangle + \frac{\varrho_5}{\mathrm{t}}\int_0^{\mathrm{t}} \breve{R}(r)d\mathfrak{D}_5(r), \end{cases} \tag{19}$$

Adding the previous equations of (19), we obtain

$$\begin{aligned} W &= \frac{\breve{S}_1(\mathrm{t})-\breve{S}_1(0)}{\mathrm{t}} + \frac{\breve{S}_2(\mathrm{t})-\breve{S}_2(0)}{\mathrm{t}} + \frac{\breve{I}_1(t)-\breve{I}_1(0)}{\mathrm{t}} + \frac{\breve{I}_2(\mathrm{t})-\breve{I}_2(0)}{\mathrm{t}} + \frac{\breve{R}(\mathrm{t})-\breve{R}(0)}{\mathrm{t}}, \\ &= \mu - \delta\langle \breve{S}_1\rangle - \delta\langle \breve{S}_2\rangle - \delta\langle \breve{I}_1\rangle - \delta\langle \breve{I}_2\rangle - \delta\langle \breve{R}\rangle - \gamma\langle \breve{I}_1\rangle - \gamma\langle \breve{I}_2\rangle - \sigma\langle \breve{I}_1\rangle - \sigma\langle \breve{I}_2\rangle \\ &+ \frac{\varrho_1}{\mathrm{t}}\int_0^{\mathrm{t}} \breve{S}_1(r)d\mathfrak{D}_1(r) + \frac{\varrho_2}{\mathrm{t}}\int_0^{\mathrm{t}} \breve{S}_2(r)d\mathfrak{D}_2(r) \\ &+ \frac{\varrho_3}{\mathrm{t}}\int_0^{\mathrm{t}} \breve{I}_1(r)d\mathfrak{D}_3(r) + \frac{\varrho_4}{\mathrm{t}}\int_0^{\mathrm{t}} \breve{I}_2(r)d\mathfrak{D}_4(r) + \frac{\varrho_5}{\mathrm{t}}\int_0^{\mathrm{t}} \breve{R}(r)d\mathfrak{D}_5(r) \\ W &= \mu - \delta\langle \breve{S}_1\rangle - \delta\langle \breve{S}_2\rangle - \delta\langle \breve{I}_1\rangle - \delta\langle \breve{I}_2\rangle - \delta\langle R\rangle - \gamma\langle \breve{I}_1\rangle - \gamma\langle \breve{I}_2\rangle - \sigma\langle \breve{I}_1\rangle - \sigma\langle \breve{I}_2\rangle + \frac{L}{\mathrm{t}}, \end{aligned}$$

where

$$\begin{aligned} \frac{L}{\mathrm{t}} &= \frac{\varrho_1}{\mathrm{t}}\int_0^{\mathrm{t}} \breve{S}_1(r)d\mathfrak{D}_1(r) + \frac{\varrho_2}{\mathrm{t}}\int_0^{\mathrm{t}} \breve{S}_2(r)d\mathfrak{D}_2(r) + \frac{\varrho_3}{\mathrm{t}}\int_0^{\mathrm{t}} \breve{I}_1(r)d\mathfrak{D}_3(r) \\ &+ \frac{\varrho_4}{\mathrm{t}}\int_0^{\mathrm{t}} \breve{I}_2(r)d\mathfrak{D}_4(r) + \frac{\varrho_5}{\mathrm{t}}\int_0^{\mathrm{t}} \breve{R}(r)d\mathfrak{D}_5(r). \end{aligned}$$

Hence

$$\begin{aligned} \delta\langle \breve{S}_1\rangle &= \mu - \delta\langle \breve{S}_2\rangle - \delta\langle \breve{I}_1\rangle - \delta\langle \breve{I}_2\rangle - \delta\langle R\rangle - \gamma\langle \breve{I}_1\rangle - \gamma\langle \breve{I}_2\rangle - \sigma\langle \breve{I}_1\rangle - \sigma\langle \breve{I}_2\rangle + \frac{L}{\mathrm{t}} \\ &+ \frac{\breve{S}_1(\mathrm{t})-\breve{S}_1(0)}{\mathrm{t}} + \frac{\breve{S}_2(\mathrm{t})-\breve{S}_2(0)}{\mathrm{t}} + \frac{\breve{I}_1(\mathrm{t})-\breve{I}_1(0)}{\mathrm{t}} + \frac{\breve{I}_2(\mathrm{t})-\breve{I}_2(0)}{\mathrm{t}} + \frac{\breve{R}(\mathrm{t})-\breve{R}(0)}{\mathrm{t}}, \end{aligned} \tag{20}$$

Substitute (20), we obtain

$$\frac{\ln \breve{I}_1(\mathrm{t})}{\mathrm{t}} \leq \frac{\beta_1}{\delta}[\mu - \delta\langle\breve{S}_2\rangle - \delta\langle\breve{I}_1\rangle - \delta\langle\breve{I}_2\rangle - \delta\langle\breve{R}\rangle - \gamma\langle\breve{I}_1\rangle - \gamma\langle\breve{I}_2\rangle - \sigma\langle\breve{I}_1\rangle - \sigma\langle\breve{I}_2\rangle$$
$$+ \frac{L}{\mathrm{t}} + \frac{\breve{S}_1(\mathrm{t}) - \breve{S}_1(0)}{\mathrm{t}} + \frac{\breve{S}_2(\mathrm{t}) - \breve{S}_2(0)}{\mathrm{t}} + \frac{\breve{I}_1(\mathrm{t}) - \breve{I}_1(0)}{\mathrm{t}} + \frac{\breve{I}_2(\mathrm{t}) - \breve{I}_2(0)}{\mathrm{t}} +$$
$$\frac{\breve{R}(\mathrm{t}) - \breve{R}(0)}{\mathrm{t}}] - \delta(\mathrm{t}) - \frac{1}{2}{\varrho_2}^2\mathrm{t} + P_1(\mathrm{t}), \tag{21}$$

Hence, utilizing study in [17] we can write

$$\lim_{\mathrm{t}\to\infty} \frac{\ln \breve{I}_1(\mathrm{t})}{\mathrm{t}} \leq \frac{\beta_1\mu}{\delta} - (\gamma + \delta + \sigma) - \frac{1}{2}{\varrho_2}^2,$$

$$\leq (\gamma + \delta + \sigma)\,[\mathbf{R}_1 - 1] - \frac{1}{2}{\varrho_2}^2,$$

then

$$\lim_{\mathrm{t}\to\infty} \frac{\ln \breve{I}_1(\mathrm{t})}{\mathrm{t}} \leq (\gamma + \delta + \sigma)\left[\mathbf{R}_1 - 1 - \frac{\varrho_2^2}{2\,(\gamma + \delta + \sigma)}\right]. \tag{22}$$

The process can be repeated to show $\lim_{\mathrm{t}\to\infty} \breve{I}_2(\mathrm{t}) = 0$ when $\mathbf{R}_2^s < 1$.

12.7 Codes

```
import numpy as np
from scipy.integrate import odeint
import matplotlib.pyplot as plt
import seaborn as sns
from mpl_toolkits.mplot3d import Axes3D
import pandas as pd
def model(y, t, beta0, alpha, r, K, gamma, mu, delta, sigma, rho):
    S1, S2, I1, I2, R = y
    I = I1 + I2
    beta1 = beta2 = beta0 * (1 + alpha * r * I * (1 - I / K))

    dS1 = mu - beta1 * S1 * I1 - beta2 * S1 * I2 + rho * R - delta * S1
    dS2 = -beta2 * S2 * I2 - beta1 * S2 * I1 - delta * S2
    dI1 = beta1 * S2 * I1 - gamma * I1 - sigma * I1 - delta * I1
    dI2 = beta2 * S1 * I2 - gamma * I2 - sigma * I2 - delta * I2
    dR = gamma * I1 + gamma * I2 - rho * R - delta * R
```

```
    return [dS1, dS2, dI1, dI2, dR]
S1_0 = 0.9
S2_0 = 0.9
I1_0 = 0.01
I2_0 = 0.01
R_0 = 0.0
y0 = [S1_0, S2_0, I1_0, I2_0, R_0]
t = np.linspace(0, 65, 5000)
beta0 = 0.5
alpha = 0.1
r = 1.0
K = 1.0
gamma = 0.1
mu = 0.02
delta = 0.01
sigma = 0.01
rho = 0.005
sol = odeint(model, y0, t, args=(beta0,alpha,r,K,gamma,mu,delta,sigma,rho))
plt.figure(figsize=(12, 8))
plt.plot(t, sol[:, 0], 'b', label='S1')
plt.plot(t, sol[:, 1], 'g', label='S2')
plt.plot(t, sol[:, 2], 'r', label='I1')
plt.plot(t, sol[:, 3], 'm', label='I2')
plt.plot(t, sol[:, 4], 'c', label='R')
plt.title('Dynamics of Susceptibles, Infected, and Recovered Populations')
plt.xlabel('Time')
plt.ylabel('Population Fraction')
plt.legend()
plt.grid(True)
plt.tight_layout()
plt.show()
##Population Distribution ##
populations_at_t100 = [sol[index_at_t100, i] for i in range(5)]
labels = ['S1', 'S2', 'I1', 'I2', 'R']
colors = ['blue', 'green', 'red', 'magenta', 'cyan']
plt.figure(figsize=(8, 8))
plt.pie(populations_at_t100, labels=labels, colors=colors, autopct='%1.1f%%')
plt.title('Population Distribution at t=100')
plt.show()
##Time series##
plt.figure(figsize=(10, 6))
plt.plot(t, sol[:, 0], label='S_1')
plt.plot(t, sol[:, 1], label='S_2')
plt.plot(t, sol[:, 2], label='I_1')
plt.plot(t, sol[:, 3], label='I_2')
plt.plot(t, sol[:, 4], label='R')
```

```
plt.legend()
plt.title('Time Series of Populations')
plt.xlabel('Time')
plt.ylabel('Population Fraction')
plt.grid(True)
plt.show()
##Phase Plane##
plt.figure(figsize=(10, 6))
plt.plot(sol[:, 0], sol[:, 2], label='S_1 vs I_1')
plt.plot(sol[:, 1], sol[:, 3], label='S_2 vs I_2')
plt.legend()
plt.title('Phase Plane Plot')
plt.xlabel('Susceptible Population')
plt.ylabel('Infected Population')
plt.grid(True)
plt.show()
##Quiver Plot###
U = sol[1:, 2] - sol[:-1, 2]
V = sol[1:, 3] - sol[:-1, 3]
plt.figure(figsize=(10, 6))
plt.quiver(sol[:-1, 2], sol[:-1, 3], U, V, scale=50)
plt.title('Quiver Plot showing changes in I1 and I2')
plt.xlabel('I1')
plt.ylabel('I2')
plt.grid(True)
plt.show()
##moving_avg##
moving_avg_I1 = np.convolve(sol[:, 2], np.ones(50)/50, mode='valid')
moving_avg_I2 = np.convolve(sol[:, 3], np.ones(50)/50, mode='valid')
plt.figure(figsize=(10, 6))
plt.plot(t, sol[:, 2], label='I1')
plt.plot(t[len(t)-len(moving_avg_I1):],moving_avg_I1,'r--',label='I1')
plt.plot(t, sol[:, 3], label='I2')
plt.plot(t[len(t)-len(moving_avg_I2):],moving_avg_I2,'m--',label='I2')
plt.title('Time Series with Moving Average')
plt.xlabel('Time')
plt.ylabel('Population Fraction')
plt.legend()
plt.grid(True)
plt.show()
```
